# Supplementary material for: G-Anchor: a novel approach for whole-genome comparative mapping utilizing evolutionary conserved DNA sequences
Source: Gigascience. 2018 Apr 3;7(5):giy017. doi: 10.1093/gigascience/giy017 (PMC5961299; doi:10.1093/gigascience/giy017)
Supplement: GIGA-D-17-00295_Revision_1.pdf [file giy017_giga-d-17-00295_revision_1.pdf]

GigaScience

G-Anchor: a novel approach for whole-genome comparative mapping utilising evolutionary conserved DNA sequences

--Manuscript Draft--

**Manuscript Number:**	GIGA-D-17-00295R1
**Full Title:**	G-Anchor: a novel approach for whole-genome comparative mapping utilising evolutionary conserved DNA sequences
**Article Type:**	Technical Note
**Funding Information:**	Biotechnology and Biological Sciences Research Council (BB/J010170/1) — Dr Denis M Larkin
**Abstract:**	Abstract<br>Background<br>Cross-species whole-genome sequence alignment is a critical first step for genome comparative analyses ranging from the detection of sequence variants to studies of chromosome evolution. Animal genomes are large and complex, and whole-genome alignment is a computationally intense process, requiring expensive high performance computing systems due to the need to explore extensive local alignments. With hundreds of sequenced animal genomes available now from multiple projects there is an increasing demand for genome comparative analyses.<br>Results<br>Here we introduce G-Anchor, a new, fast, and efficient pipeline that uses a strictly limited but highly effective set of local sequence alignments to anchor (or map) an animal genome to another species' reference genome. G-Anchor makes novel use of a databank of highly conserved DNA sequence elements. We demonstrate how these elements may be aligned to a pair of genomes, creating anchors. These anchors enable the rapid mapping of scaffolds from a de novo assembled genome to chromosome assemblies of a reference species. Our results demonstrate that G-Anchor can successfully anchor a vertebrate genome onto a phylogenetically related reference species genome using a desktop or laptop computer within a few hours, and with comparable accuracy to that achieved by a highly accurate whole-genome alignment tool such as LASTZ. G-Anchor thus makes whole-genome comparisons accessible to researchers with limited computational resources.<br>Conclusions<br>G-Anchor is a ready-to-use tool for anchoring a pair of vertebrate genomes. It may be used with large genomes that contain a significant fraction of evolutionally conserved DNA sequences, and that are not highly repetitive, polypoid or excessively fragmented. G-Anchor is not a substitute for whole-genome aligning software but can be used for fast and accurate initial genome comparisons.<br>G-Anchor is freely available via https://github.com/vasilislenis/G-Anchor
**Corresponding Author:**	Vasileios Panagiotis Eustathios Lenis, PhD<br><br>UNITED KINGDOM
**Corresponding Author Secondary Information:**	
**Corresponding Author's Institution:**	
**Corresponding Author's Secondary Institution:**	
**First Author:**	Vasileios Panagiotis Eustathios Lenis, PhD
**First Author Secondary Information:**	
**Order of Authors:**	Vasileios Panagiotis Eustathios Lenis, PhD
	Martin Swain
	Denis M Larkin

Powered by Editorial Manager® and ProduXion Manager® from Aries Systems Corporation

**Order of Authors Secondary Information:**	
**Response to Reviewers:**	We thank for the reviewers for their further feedback. Our response to their suggestions is as follows.<br><br>Reviewer 1:<br>To more easily compare the run-time metrics of LASTZ, MUMmer, and G-Anchor, please report how long a whole genome alignment of the yak and cattle genomes takes using MUMmer 4.0. Please report in the same paragraph as LASTZ and MUMmer the total run-time of all stages of the G-Anchor pipeline for mapping the Yak genome to the Cow genome. Please report the multi-threaded wall-clock run-times of MUMmer and G-Anchor, noting that LASTZ is not multithreaded.<br><br>We have added this material between lines 505 and 544. Briefly:<br>"We give the time for LASTZ noting that is not multi-threaded (5 days, 4 hours).<br>"We report that MUMmer 4.0 takes 48 hours with a single core and 12 hours with 4 cores, and requires 43 GB RAM in both cases.<br>"G-Anchor requires 7 hours with a single core and 3 hours 15 minutes for 4 cores with 4.5 GB RAM. For the single core case, the times for the five stages are 4, 12, 11, 353, and 43 minutes respectively. Aligning the HCE with Blat takes the longest time: this was reduced from 353 (1 core) to 124 minutes (4 cores).<br><br>Reviewer 2:<br>Minor comments: 1. Combining math and (pseudo)code (e.g., Page 19 equation), while may serve the purpose of conveying the notion, may be written clearer using a standard pure math or pure algorithm notation.<br><br>We have added a standard pure math notation of the equation, between lines 436 to 444, as requested.<br><br>Editor:<br>In addition, please register any new software application in the SciCrunch.org database to receive a RRID (Research Resource Identification Initiative ID) number, and include this in your manuscript. This will facilitate tracking, reproducibility and re-use of your tool.<br><br>We have registered the software and added the RRID number (SCR_016046) at line 666.
**Additional Information:**	
**Question**	**Response**
Are you submitting this manuscript to a special series or article collection?	No
**Experimental design and statistics**<br><br>Full details of the experimental design and statistical methods used should be given in the Methods section, as detailed in our Minimum Standards Reporting Checklist. Information essential to interpreting the data presented should be made available in the figure legends.<br><br>Have you included all the information requested in your manuscript?	Yes
**Resources**<br><br>A description of all resources used,	Yes

including antibodies, cell lines, animals and software tools, with enough information to allow them to be uniquely identified, should be included in the Methods section. Authors are strongly encouraged to cite Research Resource Identifiers (RRIDs) for antibodies, model organisms and tools, where possible.<br><br>Have you included the information requested as detailed in our Minimum Standards Reporting Checklist?	
**Availability of data and materials**<br><br>All datasets and code on which the conclusions of the paper rely must be either included in your submission or deposited in publicly available repositories (where available and ethically appropriate), referencing such data using a unique identifier in the references and in the “Availability of Data and Materials” section of your manuscript.<br><br>Have you have met the above requirement as detailed in our Minimum Standards Reporting Checklist?	Yes

Powered by Editorial Manager® and ProduXion Manager® from Aries Systems Corporation

Manuscript
Click here to download Manuscript G-Anchor_resubmission-Final_Version13-2.docx

G-Anchor: a novel approach for whole-genome comparative mapping utilising evolutionary conserved DNA sequences

Vasileios Panagiotis E. Lenis[1,2], Martin Swain[1]* and Denis M. Larkin[3]*

[1]Institute of Biological, Environmental and Rural Sciences, Aberystwyth University, Aberystwyth, SY23 3DA, UK

[2]School of Biomedical and Healthcare Sciences, Plymouth University Peninsula, Schools of Medicine and Dentistry, Plymouth PL6 8BU

[3]Department of Comparative Biomedical Sciences, Royal Veterinary College, Royal College Street, University of London, London, NW1 0TU, UK

*Correspondence to: Denis M. Larkin (dmlarkin@gmail.com) and Martin T. Swain (mts11@aber.ac.uk)

Keywords: whole-genome alignment, sequence mapping, sequencing anchoring, Highly Conserved Elements (HCE), genome evolution.

Abstract

Background

Cross-species whole-genome sequence alignment is a critical first step for genome comparative analyses ranging from the detection of sequence variants to studies of chromosome evolution. Animal genomes are large and complex, and whole-genome alignment is a computationally intense process, requiring expensive high performance computing systems due to the need to explore extensive local alignments. With hundreds of sequenced animal genomes available now from multiple projects there is an increasing demand for genome comparative analyses.

Results

Here we introduce *G-Anchor*, a new, fast, and efficient pipeline that uses a strictly limited but highly effective set of local sequence alignments to anchor (or map) an animal genome to another species' reference genome. G-Anchor makes novel use of a databank of highly conserved DNA sequence elements. We demonstrate how these elements may be aligned to a pair of genomes, creating anchors. These anchors enable the rapid mapping of scaffolds from a *de novo* assembled genome to chromosome assemblies of a reference species. Our results demonstrate that G-Anchor can successfully anchor a vertebrate genome onto a phylogenetically related reference species genome using a desktop or laptop computer within a few hours, and with comparable accuracy to that achieved by a highly accurate whole-genome alignment tool such as LASTZ. G-Anchor thus makes whole-genome comparisons accessible to researchers with limited computational resources.

Conclusions

G-Anchor is a ready-to-use tool for anchoring a pair of vertebrate genomes. It may be used with large genomes that contain a significant fraction of evolutionally

conserved DNA sequences, and that are not highly repetitive, polypoid or excessively fragmented. G-Anchor is not a substitute for whole-genome aligning software but can be used for fast and accurate initial genome comparisons.

G-Anchor is freely available via https://github.com/vasilislenis/G-Anchor

Findings

1. Introduction

Accurate alignment of two or more genomes is an important step for applications such as annotating a *de novo* sequenced and assembled genome, performing cross-species genome evolutionary studies, reconstructing ancestral genomes [1]–[3] and for detecting variations and genes under selection within a species [4]. Unfortunately, the whole-genome alignments of large genomes (such as animal genomes over 1 Gb in size) with most contemporary alignment algorithms require significant computational resources and therefore imply the use of high performance computing (HPC) systems containing hundreds of CPUs and dozens of gigabytes of RAM [5]. Such systems are expensive and often are not available to a smaller laboratory or research group. On the other hand, the progress recently achieved in high-throughput sequencing technologies makes the sequencing of a complex genome a relatively trivial and non-expensive endeavor [6]. As a result, more than a hundred mammalian, avian and other animal whole-genome assemblies are already available from genome repositories or private databases [5]. Hundreds more genomes are currently being sequenced by the Genome 10K community [7], other international consortia or by individual groups [8], [9]. Many of these genomes are being included in bulk annotations produced by large

genomic centers, and multiple whole-genome alignments are publically available from centralized databases like Ensembl or the UCSC Genome Browser [10], [11]. However, other genome assemblies, such as those produced by smaller research groups, may not be represented in public databases and are therefore excluded from these bulk comparisons and related bioinformatics resources. As a result, the comparative analyses that may be performed on these genomes are limited. In this paper we introduce software for whole-genome anchoring that aims to address some of these issues.

The analysis of multiple whole-genome alignments demonstrates that animal genomes contain a significant fraction of highly conserved elements (HCE). Evolutionary pressures are thought to conserve HCE, which are comprised of gene coding sequences, non-coding regulatory elements, or evolutionary stable DNA sequences with a structural role (e.g., the lamina-associated DNA) [12]–[14]. These elements range from 1 base pair (bp) to about several hundred base pairs and represent ~5% of a mammalian genome or ~15% of an avian genome [15]. If genome alignments are further limited to phylogenetically close species (within an Order or family) the fraction of HCE increases to ~15% for mammals and ~20% for birds [16]. In the context of analyses based on cross-species comparisons, conserved sequences are naturally occurring land marks in a DNA sequence that are stable over relatively large evolutionary times.

Here we propose to use HCE as "anchors" for fast low-pass alignments of genome assemblies. Instead of a full pairwise alignment between two genomes derived from comprehensive and time-consuming local alignments, our anchoring approach is able to use HCE to quickly generate a limited but effective set of local alignments. These HCE alignments (or anchors) are able to predict the location in

which the scaffolds of a newly sequenced genome would be placed if a whole-genome alignment were performed. We believe this work is the first to explore the use of HCE as anchors in comparative genomics applications. As a result, G-Anchor has the potential to open up whole genome comparisons of vertebrate genomes to a much wider set of researchers. In our opinion, G-Anchor is unique in this aspect: it is the only tool currently available that allows whole vertebrate genome comparisons to be made on a simple personal computer. In addition, to aid downstream analyses, G-Anchor creates output suitable for use with the suite of visualization and other tools available on the widely used UCSC Genome Browser. G-Anchor does not aim at achieving the resolution and completeness of whole-genome pairwise alignments built with traditional whole-genome alignment tools (like LASTZ or MUMmer) [17], [18] but provides a fast and sensitive way of anchoring two large genomes using more accessible computational resources i.e. a desktop workstation or laptop.

In the following sections, we explain the G-Anchor algorithm and how it has been implemented in a pipeline. We explain how sets of HCE from pre-existing alignments may be readily used in the pipeline, or alternatively if desired, how customized sets of HCE may be generated from bespoke multiple whole-genome alignments. The results of G-Anchor are evaluated using a number of test-cases: for instance, we compare the G-Anchor predicted order of scaffolds to LASTZ-based whole-genome alignments, and quantify the significantly reduced computational resources required by G-Anchor.

2. Data preparation and preprocessing

Here we explain how sets of HCE may be generated, either by generating the multiple whole-genome alignment, or from a pre-existing multiple alignment. Then we describe how a set of HCE may be processed to generate an HCE databank to be used with G-Anchor. Finally, we describe the construction of a “mammalian” HCE databank that may be used with a wide range of mammals.

Constructing multiple whole-genome alignments

Highly conserved element (HCE) datasets may be generated via pairwise whole-genome alignments created using LASTZ (version 1.02.00). Here, (for datasets Cow+Yak, Cow-Yak, described fully in Section 4) we used LASTZ with the following parameters: *E=30, H=2000, K=3000, L=2200, O=400* and the default substitution matrix. The alignments were post-processed into the UCSC Genome Browser *chain* and *net* data formats, which are higher-level abstractions of pairwise sequence alignments. A *chain* represents an ordered sequence of the alignments, separated by regions lacking alignments (gaps). On the other hand, a *net* constitutes a hierarchy of chains where the chains with the lower scores fit within the gaps present in the highest scoring chain [19]. Chains and nets were constructed with tools from the Kent’s toolbox (version v349) [11] with the following parameters for chain and net construction: *-verbose=0 -minScore=3000 and -linearGap=medium/loose* (the “medium” value was used for the net construction of LASTZ based alignments and the “loose” for the G-Anchor mapping process). From chains and nets, the multiple alignment format (MAF) files were finally built with MULTIZ [20], also using the phylogenetic relationships and distances between species in our dataset (Figure 1).

Extracting HCE from multiple whole-genome alignments

Once MAF files are produced, the coordinates of the HCE may be defined. The reference based sequence coordinates of the HCE were identified with *phastCons* [13] using the appropriate set of parameters for each dataset (see Additional file 1, Table 1) and applying a non-conserved phylogenetic model built by *phyloFit* [13]. This model was based on the fourfold degenerate sites (4d) from a FASTA file containing the gene coding regions (CDS) obtained from the UCSC Genome Browser. The sequence coordinates of all HCE in the MAF reference genome were then used to extract the actual corresponding DNA sequences from the MAF reference genome. This was performed with a minimum length of 40 bp using *fastaFromBed* tool from the *bedtools* suite [21].

Please note that the protocol for extracting HCE is well documented online at [http://genomewiki.ucsc.edu/index.php/Whole_genome_alignment_howto].

Finally, the same process was followed for the generation of an Avian HCE set. A full description of all the parameters that was used can be found in [22].

Generating an HCE databank for the G-Anchor pipeline

To ensure optimal performance of the G-Anchor pipeline for every pair of genomes compared, it is important to choose appropriate HCE from within the set of HCE coordinates extracted from a MAF file. These HCE that map with appropriate criteria to the reference genome used in the anchoring procedure are known as the *HCE databank* and are defined for a specific reference species.

Note that the G-Anchor reference genome is often different to the reference genome used to create the MAF file. In fact, the G-Anchor reference genome does not need to

be any of the genomes included in the multiple alignment MAF file from which the initial set of HCE are extracted but for optimal performance they should belong to the same clade (e.g. Class or Order). For clarity, we will refer to the G-Anchor reference genome as the *ga-reference.* The *ga-target* genome is the genome that is being anchored to the ga-reference.

All extracted HCE sequences are aligned to the ga-reference using BLAT (v. 36X1, standalone version) [23] with default alignment parameters. BLAT has been chosen because it is a fast aligner for relatively short sequences with a high level of identity. Potentially, other mapping tools could be used, such as BWA-MEM [24] and Minimap,[25] which are designed for mapping sequence reads to the same species reference genome. However, BLAT gives more flexibility in terms of the minimum percentage identity of the alignment. In addition, BLAT's alignment output, in PSL format, is more informative about the alignment blocks and is required by other components of our pipeline. Moreover, BLAT was faster than Minimap for the mapping of Yak scaffolds onto cattle autosomes in our tests (Additional file, Figure 6).

The alignment of the HCE to the ga-reference ensures that the only HCE in the databank are those with unique alignments to the ga-reference (even if the chosen ga-reference genome is different to the MAF reference genome, or if it is not included in the genomes used to create the MAF file). The resulting alignments are stringently filtered, based on 100% sequence identity and alignment length criteria of at least 99% of the HCE length. Filtering of HCE suitable for a specific ga-reference is controlled by the script *G-Anchor_preProcessing.sh*. Finally, HCE aligning to multiple locations are removed, leaving only those with a single

alignment position in the ga-reference. These remaining HCE then comprise the ga-reference-specific HCE databank (shown in Figure 2).

Preparation of an HCE data set for mammals

Using a pre-computed MAF file (i.e. that of 99 vertebrate genomes from the UCSC Genome Browser, see datasets Hum+Rum and Hum+Mam described fully in Section 4) we designed a set of HCE sequences that were present in the genomes of representatives of four Orders of mammals (ruminants, carnivores, primates and rodents). These elements were identified from the four Orders using the human genome as reference and then combined in a single file. We intentionally did not remove any elements from this HCE data set that had overlapping coordinates in the MAF reference genome (human). This ensures that the longest HCE with the highest quality alignment is available to G-Anchor, no matter what ga-reference genome was used. An HCE databank is defined for a particular species once the mammalian HCE data set is mapped against a ga-reference.

3. G-Anchor pipeline

The G-Anchor pipeline combines several published tools (including BLAT, and 10 scripts from Kent stand-alone tools [11]), as well as 13 novel Perl scripts. The G-Anchor Perl scripts are controlled using two Bash shell scripts called *G-Anchor.sh* and *G-Anchor_controller.sh*. The *G-Anchor.sh* script processes all input files including the ga-reference and ga-target genome sequences, the ga-reference-specific HCE databank, and the numbers of the ga-reference genome's chromosomes. The *G-Anchor_controller.sh* script was designed to work in an interactive way in order to

process the user-defined arguments and to report to the user possible errors in the command line arguments or in the input file structures.

The G-Anchor pipeline consists of five major stages: 1) Preprocessing of the ga-reference and ga-target genomes; 2) Aligning the ga-reference-specific HCE databank against the ga-target genome; 3) Filtering of HCE to construct anchors on the ga-target; 4) Transferring the constructed anchors onto the ga-reference; 5) Construction of chains and nets on the ga-reference that define the mapping between the two genomes. A complete G-Anchor workflow is shown in Figure 2.

Stage 1: Preprocessing of the ga-reference and ga-target genomes

G-Anchor inputs are the ga-target and ga-reference genomes, both stored in a binary (2bit) format, with the ga-reference assembled in chromosomes or pseudo-chromosomes; and the HCE databank (described in section 2 and computed for the ga-reference). There is no specific restriction on the contiguity of the ga-reference assembly, but a highly fragmented ga-reference assembly (assembled in many scaffolds rather than chromosomes), could dramatically increase G-Anchor's running time. The ga-reference and ga-target genome files are stored in the same "GENOMES" folder (separate "Reference" and "Target" subfolders) while the HCE databank is stored in a separate folder using a multi-FASTA format. During the preprocessing step the *G-Anchor.sh* script generates the output folders and converts both input files into the multi-FASTA format with the *twoBitToFa* tool. The sizes (in base pairs) of the chromosomes or scaffolds in both of the genomes are then calculated with the *faSize* tool. Both the FASTA sequences and files output from *faSize* are stored in a temporary folder, which is automatically deleted after the end of the G-Anchor run.

Stage 2: HCE databank alignment against the ga-target genome

All HCE from the HCE databank are aligned against the ga-target using BLAT with the default minimum sequence identity 90%. The alignment process is the most time-consuming stage of the G-Anchor pipeline. Therefore, we allow the user to choose from several BLAT command line options that can speed up the alignment. These options include *-ooc* or *-fastMap* parameters that either decrease the running time by excluding over-represented sequences from the seeding stage (*-ooc*), or that skip the time-consuming stage of merging alignment blocks that have gaps between them (*-fastMap*). In addition, G-Anchor provides options to run the alignment stage using multiple cores. The G-Anchor default alignment process uses a single core with none of the parameters enabled.

Longer HCE may not align as efficient as shorter HCE because of higher chances of containing mismatches or gaps in alignments, and so they may disproportionally fail to pass the G-Anchor alignment filters. They can also consume significant time for their alignment and cause conflicts when *–fastMap* option is enabled (maximum sequence length that *–fastMap* can handle is 5 Kb). Therefore, we provide an option to split longer HCE into shorter sequences, which align better and faster to the reference and target genomes. The splitting option is automatically activated to split the HCE that are larger than 5 Kb when *–fastMap* option is chosen.

In addition, G-Anchor provides another two parameters that give the ability to relax the alignment criteria of the HCE, suitable for more distant genomes (See "G-Anchor in more divergence sequences" section). *–minIdentity* and *–minAli* allow the control of the minimum similarity identity and the minimum percentage sequence identity (later is described in the following stage), respectively (Additional file 1, Figures 3-

5).

Stage 3: Filtering the alignments of the HCE databank against the ga-target

BLAT identifies all the ga-target intervals where an HCE aligns or partially aligns. Since the HCE (by definition) are sequences having a high level of conservation across multiple genomes [26] the alignment criteria can be stringent. G-Anchor scripts parse the BLAT alignments to find the intervals where HCE align with high percentage sequence identity across HCE length (typically around 95%, but as low as 80% is possible) to the ga-target and use only HCE meeting this criterion for the next step. These HCE are sorted by their alignment positions in the ga-target, and then they are further filtered by removing HCE that map to multiple positions in the ga-target. Only these HCE are used by G-Anchor as markers for anchoring the ga-reference and ga-target sequences: these HCE map uniquely to both the ga-reference and the ga-target and are now called *HCE anchors*. Finally, these HCE anchors are stored and used for cleaning the initial alignment files in PSL format by preserving only the HCE databank alignments that include HCE anchors.

STAGE 4: Post processing of the aligned HCE anchors

To anchor, order and orient the ga-target scaffolds in the ga-reference, G-Anchor utilizes functionality of the UCSC Genome Browser relating to the chain and net data formats. The chains and nets are constructed between the locations of the two genomes where the HCE anchors align. (Note that each HCE anchor also has unique ga-reference coordinates, identified during the HCE databank construction). The correspondence of the two sets of coordinates is used by G-Anchor to identify the

correspondence between the intervals of the ga-target and the ga-reference. To build the chains and nets from alignments of HCE anchors, the anchors need to be transferred from the ga-target PSL file onto the ga-reference. This is performed using the program *pslSwap* to create a PSL file for the ga-reference.

STAGE 5: Chains and nets construction

The ga-reference PSL file is required as input for the UCSC Genome Browser chains and nets construction pipeline. Chains are built with the UCSC Genome Browser *axtChain* tool [19] and link multiple locations where HCE align as anchors. The second level merging into nets is achieved with *chainNet.* It generates a hierarchical collection of the longer, higher-level non-overlapping chains, filling their gaps (if possible) with the shorter, lower-level chains – essentially, it combines chains into longer alignment constructs. Finally, *netSyntenic* is used to add information on the relationship of continuous two level chains in the nets file. For the G-Anchor's nets construction the "loose" value was used.

The final G-Anchor outputs include: a) the gapless alignments of the ga-reference and ga-target genomes restricted to the HCE anchor intervals, in PSL format; b) the longer chain and net alignment constructions of the ga-reference and ga-target; c) a textual report file with numbers of HCE that support the anchoring of each ga-target genome sequence to the ga-reference genome.

4. Testing and Evaluation

Defining the ga-reference, ga-target, and the HCE databanks

The G-Anchor pipeline was tested intensively in a number of different cases such as:

a) genomes of closely related species (mammalian genomes); b) genomes from a different class of species (avian genomes) and c) genomes of species with large sequence diversity (murid rodent genomes). Since the most interesting/common scenario is the identification of similarities in closely related species, case a) is presented more extensively in the rest of the paper. Nevertheless, the reader can find more details of the rest of the cases (b and c) in the Additional file 1.

Case a) was represented by mammalian genomes, using the scaffold assembly of the yak genome (*Bos grunniens, Yak_1.1*) as ga-target and the cattle autosomes (*Bos taurus*, *bosTau7*) as ga-reference (to create the corresponding HCE databanks). For this comparison different HCE data sets were used in separate G-Anchor mapping experiments. Two of the HCE data sets were defined using the cattle genome as the MAF reference aligned with several other ruminant genomes. Two other HCE data sets used the human genome as the MAF reference, aligned with combinations of ruminant and non-ruminant genomes. In order to examine how G-Anchor performs on avian genomes (case b), we applied the pipeline using the scaffold assembly of the Mallard duck genome (*Anas Platyrhynchos*, *BGI_duck_1.0*) as ga-target and the chicken autosomes (*Gallus gallus*, Galgal4) as ga-reference. Finally, G-Anchor was tested further in the comparison of genomes with high sequence diversity as human and mouse (*Mus musculus*, mm10) genomes (case c). The mouse genome was anchored onto human's autosomes by using the mammalian HCE data set, which is described below. Details can be found in Additional file 1 (Table 4, Figures 3 - 5).

Five different HCE datasets and databanks were generated as follows:

1. *Ruminant data set including yak with cattle as the ga-reference;* **Cow+Yak**: was used to define the most complete HCE data set shared by ruminant genomes. This set included the Tibetan antelope (*Pantholops hodgsonii, panHod1*), sheep

(*Ovis aries, oviAri3*), goat (*Capra hircus*, *capHir1*) and yak (*Bos grunniens, Yak_1.1*) genome assemblies aligned against the cattle genome.

2. *Ruminant data set excluding yak with cattle as the ga-reference*; **Cow-Yak**: was used to test the effect of excluding the ga-target genome from the multiple alignment when creating the HCE data set. This set includes the Tibetan antelope, sheep, and goat assemblies aligned against the cattle genome.
3. *Ruminant genomes aligned against the human genome with cattle as the ga-reference;* **Hum+Rum**: was used to test the effect of creating the HCE data set using a pre-existing multiple alignment that is based on pairwise alignments to a single reference genome (human, *Homo sapiens, hg38*), and which includes 99 species from more than one taxonomic order. All species were removed, except for Tibetan antelope, sheep and goat genomes. These genomes were originally aligned pairwise against the human genome, which in this case is evolutionarily distant from the ga-reference (cattle).
4. *Mammalian HCE data set with cattle as the ga-reference;* **Hum+Mam**: was used to test the effect of creating the HCE data set (for any potential ga-reference mammalian species) using a pre-existing multiple alignment that is based on pairwise alignments to a single reference genome (human, *Homo sapiens, hg38*), and which includes 99 species from more than one taxonomic order. The mammalian HCE data set includes representatives of Primates: *Pan troglodytes* (*panTro4), Gorilla gorilla* (*gorGor3), Pongo pygmaeus abelii* (*ponAbe2), Nomascus leucogenys* (*nomLeu3), Macaca mulatta* (*rheMac3), Macaca fascicularis* (*macFas5), Papio hamadryas* (*papAnu2), Chlorocebus sabaeus* (*chlSab2), Callithrix jacchus* (*calJac3), Saimiri* boliviensis (*saiBol1), Otolemur garnettii* (*otoGar3)*; Rodents: *Spermophilus tridecemlineatus* (*speTri2), Jaculus jaculus* (*jacJac1),*

Microtus ochrogaster (*micOch1*), *Cricetulus griseus* (*criGri1*), *Mesocricetus auratus* (*mesAur1*), *Mus musculus* (*mm10*), *Rattus norvegicus* (*rn6*), *Heterocephalus glaber* (*hetGla2*), *Cavia porcellus* (*cavPor3*), *Chinchilla lanigera* (*chiLan1*), *Octodon degus* (*octDeg1*), *Oryctolagus cuniculus* (*oryCun2*), *Ochotona princeps* (*ochPri3*) and Carnivores*: Felis catus (felCat8), Canis lupus familiaris (canFam3), Mustela putorius furo (musFur1), Ailuropoda melanoleuca (ailMel1)*.

5. *Avian HCE data set with chicken as the ga-reference*; **Avian**: was used to define the most complete HCE data set shared by 20 avian genomes. Details about the genomes that were used for the HCE data set can be found in the Additional file 1 (section 4). Here mallard was used at the ga-target.

Evaluation of the HCE data sets and databanks

Before evaluating the performance of G-Anchor, we first analyze the HCE databanks, and the HCE data sets extracted from the multiple alignments used to create the databanks. As explained in Section 2, the HCE databanks are sets of HCE aligned to unique positions on the ga-reference's autosomes. More than 99% of HCE (ratio of uniquely mapped to mapped HCE in Table 1, Cattle MAF-reference) originating from either the Cow+Yak or Cow-Yak HCE data sets aligned uniquely to cattle autosomes during the preprocessing step (Preprocessed HCE on Fig. 2) and covered 16% of the cow genome. This compares to 35% (275,923 / 793,064, Table 1) of the Hum+Rum and 17% (2,139,902 / 360,322, Table 1) of Hum+Mam HCE uniquely aligning to cattle autosomes covering 2% of the ga-reference each (Table 1). As a result, in Cow-Yak we had 853,348 HCE uniquely aligned to cattle autosomes, representing the size of the HCE databank or potential HCE anchors;

and for the Hum+Rum and Hum+Mam HCE datasets, 275,924 and 360,322 potential HCE anchors, respectively.

Figure 3 shows that the number of the potential HCE anchors was similar for all 4 databanks when the potential anchors were 100-199 bp long (i.e. around 120,000 to 140,000), although longer anchors (199 bp or more) were more common in Cow+Yak and Cow-Yak databanks. When comparing Cow+Yak to Cow-Yak, both databanks provided much the same numbers of HCE (Fig 3a). However, when comparing the Hum+Mam to Hum+Rum, the former provided 23.4% (fraction of the difference between Hum+Mam and Hum+Rum HCE uniquely mapped, Table 1) more potential anchors for all length categories (Fig 3b).

To maximise the number of HCE aligning and to increase G-Anchor's time performance, we used the appropriate option in G-Anchor to break long HCE originating from the data set used to create the Hum+Mam databank. In Figure 4 the highest fraction of unbroken HCE lengths that successfully align corresponds to a length of 200-299 bp. Using this length range as a guide, we split HCE longer than 500 bp into fragments of 250 bp. This resulted in 35,597 additional potential anchors covering the cattle genome intervals not covered by the original Hum+Mam databank.

Comparison of G-Anchor mapping results with the LASTZ-based alignments

G-Anchor's performance in terms of mapping quality was evaluated by comparing its mapping results with the alignment results of a whole-genome aligner. For this cause, LASTZ was chosen due to its high alignment accuracy and its compatibility with the UCSC chains and nets, a process that G-Anchor follows at its last stage. For this comparison we looked at:

a) The total number of target scaffolds mapped or aligned; b) the fraction of the target genome covered; c) the ordering of the scaffolds; d) the fraction of scaffold bases that were present within the net blocks resulting from the G-Anchor mapping and the LASTZ-based net blocks; e) assignment inconsistencies between the two approaches; f) the amount of required computational resources.

Number of mapped scaffolds

In these analyses we used all the scaffolds in the yak genome that are longer than 10 Kb: an initial set of 4,282 scaffolds. Of these scaffolds 3,550 were successfully aligned by LASTZ and found in LASTZ nets (Table 2). Using Cow+Yak and Cow-Yak, G-Anchor attempted to map the same initial set of yak scaffolds: in comparison to LASTZ it successfully mapped 89% of the ga-target scaffolds found in the LASTZ nets (Table 2). The difference between G-Anchor runs with these two databanks was 29 scaffolds that mapped only when Cow+Yak was used and an additional 45 scaffolds mapped by Cow-Yak only (Table 3). G-Anchor mapped 2,923 (82%) scaffolds when Hum+Rum was used and 3,012 (85%) using Hum+Mam (Table 2). Breaking long HCE for the databank (Hum+Mam) increased the number of mapped scaffolds by three (Table 2). G-Anchor mapped only those from the initial set of 4,282 yak scaffolds that were also present in the LASTZ nets. The majority (94-96%) of scaffolds that were not mapped by G-Anchor but were found in the LASTZ nets were less than 1Mb in length with their N50 being approximately two times shorter than that of the mapped scaffolds (see Additional files 2-6). G-Anchor with Cow+Yak and Cow-Yak outperformed Hum+Rum and Hum+Mam in terms of the number of scaffolds mapped to each cow autosome (Figure 5); 148 and 237, respectively, more scaffolds were mapped with the Cow-Yak databank (Table 2).

Fraction of the yak genome mapped

The total length of the yak scaffolds found in LASTZ nets was 2.535 Gb (Table 2). The total length of the yak scaffolds mapped to cattle autosomes by G-Anchor using different HCE databanks ranged from 96% to 97% of the combined length of all scaffolds aligned by LASTZ. There was 4 Mb difference between the total length of scaffolds mapped when Cow+Yak and Cow-Yak were used by G-Anchor with Cow+Yak producing a slightly longer total length. When Hum+Rum and Hum+Mam were used by G-Anchor the total length of mapped scaffolds was <30 Mb shorter than for Cow+Yak and Cow-Yak, with Hum+Mam giving an 11 Mb longer total length than Hum+Rum (Table 2).

Fraction of homologous blocks

To evaluate the agreement between the aligned regions of the G-Anchor and LASTZ-based methods, the intersecting fraction F of the homologous blocks in the nets was calculated using the following formula:

$$F = \sum_{1}^{n} \frac{C}{Z}$$

Where the sum is over the total number n of mapped scaffolds of the target genome, C is the length of the homologous blocks in the scaffold common to both G-Anchor and LASTZ, and Z is the length of the homologous blocks in the scaffold as determined by LASTZ nets only.

In terms of intersecting fraction, no significant difference was noticed between Cow+Yak and Cow-Yak (Additional file 1, Figure 1). The intersecting fraction for Hum+Rum was low (16.7%) compared to the others (76.5% in Cow+Yak and 77.2% in Cow-Yak, 77.6% and 79.9% in Mammalian and Mammalian-split, respectively), this is due to this specific dataset being built from very few species (ruminants only) in the multiple alignments with human as the reference. Nonetheless, this did not affect the anchored fraction of the yak genome (Total length of anchored scaffolds, Table 2). Despite the Mammalian datasets (split or not) were built by using the same multiple alignments (but included much more species), the large number of HCE that were aligned onto cattle genome allowed the intersecting fraction with the LASTZ-based method results to reach a higher level (see Additional file 1, Figure 1).

Mapping inconsistencies between G-Anchor and LASTZ nets

We found no serious mapping inconsistencies between G-Anchor and LASTZ results for instance yak scaffolds that map to completely different ga-reference autosome. However, there were a few partial inconsistencies: for instance in the LASTZ nets a yak scaffold alignment could be split across two ga-reference chromosomes, whereas G-Anchor would map the same scaffold to only one of these chromosomes. Table 2 shows that the number of such cases ranged from 12 scaffolds for Hum+Mam to 16 scaffolds found when Cow-Yak was used. In all cases G-Anchor seemed to miss a shorter part of a scaffold that aligns to a separate ga-reference chromosome than the rest of the scaffold. A schematic representation of such discrepancies is shown in Fig. 6.

Several additional inconsistencies (10 in total across all four cattle databanks) include cases where G-Anchor and LASTZ nets map scaffolds to the same ga-reference chromosome, and to the same position within that chromosome, but differ in the exact alignment block ends for two adjacent scaffolds. These discrepancies were found when the adjacent scaffold alignments to the ga-reference chromosome overlap each other, and the net construction pipeline scored the overlapping parts of the alignments differently for LASTZ and G-Anchor. In all cases the remaining parts of the overlapping alignment could be found at the lower level of the alignment nets (Additional file 1, Figure 2).

G-Anchor in a different class of species (bird genomes)

The HCE that were predicted initially for the Avian databank were approximately 1.4 million with median length 43 bp and covered the 10.2% of the chicken genome (Additional file, Table 2). After aligning to the ga-target (mallard) and filtering, as described in G-Anchor's stage 3, roughly 950 thousand HCE aligned to the reference genome in unique positions with a 59 bp median length and 9% genome coverage, setting the HCE anchors (Additional file, Table 2). G-Anchor pipeline managed to map a little bit less than 90% of Mallard genome's scaffolds compared to the LASTZ-based alignments, covering the 96% of LASTZ alignment blocks' length (Additional file, Table 3). The scaffolds that were not mapped are mostly small in length (less than 1Mb). The inconsistencies that were noticed (scaffolds that were mapped in a different chromosome) were 1.6% of the total number of scaffolds (Additional file, Table 3).

G-Anchor applied to genomes with divergent sequences (human to mouse comparison)

Using the same high percentage sequence identity (>95%) for the HCE filtering that was used in the closely related species, the mapping coverage that was obtained was 35% with a number of HCE anchors that was reaching roughly the 4% of the Hum+Mam databank (Additional file 1, Table 4). Relaxing BLAT's minimum similarity identity *(-minIdentity* = 80%) and the minimum percentage sequence identity *(-minAli* = 80%) for the ga-target (in Stage 3); the number of HCE anchors was increased to 18% (Additional file 1, Table 4). Consequently, G-Anchor managed to increase the mapping coverage to 88.90% (Additional file 1, Table 4).

Time and computational resources taken by G-Anchor pipeline

The alignment of the yak and cattle genomes (control "LASTZ-based alignment") was performed on a Sandybridge cluster with 200 cores, provided by HPC Wales and took 7,440 minutes (5 days and 4 hours) and 40 GB of RAM in total. Note that because the LASTZ aligner is not multithreaded, it required an extra effort to split the genome into multiple fragments and distribute them over the available cores. MUMMER 4.0 was much faster than LASTZ but still demanding in terms of memory consumption. The alignment took 2,864 minutes (48 hours) on a single core and 43 GB of RAM. In a multithread mode by using 4 cores, the time reduced significantly to 12 hours with no change to RAM required (43 GB). These tools are clearly not feasible for running on a contemporary personal computer. In contrast, the G-Anchor pipeline may be run under Linux on a desktop machine. Here tests were performed using a four CPU core Intel based system with 16 GB of RAM (of which only 4.5 Gb of RAM were required). When using parameters (described below) to

optimize the execution time and using a single core, G-Anchor required 420 minutes (7 hours), which was reduced to 194 minutes (3 hours, 14 minutes) with 4 cores. To breakdown the times for each stage in the single core case, the fastest steps are preprocessing of the genomes (Stage 1) at 4 minutes, the HCEs filtering (Stage 3) at 12 minutes, and the alignment post processing (Stage 4) at 11 minutes. The HCE databank alignment (Stage 2) needed 353 minutes (5 hours and 43 minutes) and the construction of chains and nets (Stage 5) needed 43 minutes.

The most computationally intensive part of G-Anchor is using BLAT to align HCE against the ga-target genome (Stage 2). To optimize the G-Anchor execution time we incorporated the BLAT alignment optimization parameters: *-ooc* and *-fastMap*. Using a single core with the default G-Anchor parameters, the most computationally intensive execution of G-Anchor was Cow-Yak and required around 7,200 minutes (5 days) and 4.5 GB of RAM for completion. When applying the *-ooc* option the overall time was reduced from 7,200 minutes to around 1,980 minutes (1 day and 9 hours), with the *-fastMap* option to 780 minutes (13 hours), and with both options at the same time to 420 minutes (7 hours) (Table 4). When executing G-Anchor in a parallel fashion [27] (using all four CPU cores) and with both BLAT optimization parameters, HCE alignment was reduced from 353 minutes (single core) to 124 minutes. Altogether, G-Anchor with the most computationally intensive dataset (cow-yak) required 194 minutes (3 hour and 14 mins), while the Hum+Mam dataset required 85 minutes (1 hour and 15 mins). The times required for each ga-reference chromosome, for single and multiple cores and different BLAT optimization parameters are shown in Figure 7. It is worth noting that using these BLAT parameters had little effect on the mapping results (11 fewer scaffolds were mapped with both optimization parameters included) and the fraction of the yak

genome mapped was similar i.e. 96.51% vs. 96.80% (Table 4). With Hum+Mam, by using the *-fastMap* option, G-Anchor lost 11 scaffolds, reducing the fraction of the mapped yak genome by 0.6% (Table 5). Finally, G-Anchor stages 1, 3, 4 and 5 all run on a single core and currently cannot be further optimized.

If the HCE preprocessing pipeline is used to create an HCE databank during the anchoring process, then G-Anchor's total running time increases. For instance, with the mammalian HCE data set (Hum+Mam), to use cattle as the ga-reference the total preprocessing time depends on the BLAT optimization parameters and number of cores used, as shown in Table 6: 100 minutes by using 4 cores, and 288 minutes on a single core. Using the *-ooc* parameter the running time dropped to 72 minutes and 188 minutes), respectively. Using the *-fastMap* option in addition, the preprocessing time was decreased further (55 minutes for 4 cores and 140 minutes for a single core) but with the price of losing roughly the half of the HCE, making the *-fastMap* option not suitable for this stage. Hence to optimize the performance of BLAT in the preprocessing step it is only possible to use the *-ooc* parameter.

5. Discussion

This study describes a new whole-genome mapping pipeline called G-Anchor, which allows rapid comparative anchoring of two sequenced genomes of an animal genome size (>1 Gb) from different species with the use of inexpensive computational resources such as a personal computer. Our results demonstrate that G-Anchor is capable of mapping a mammalian genome (yak) to another mammalian genome autosomes (cattle) on a personal computer in just over 3 hours and with 4.5 GB RAM, which compares to 124 hours required for a 'traditional' whole-genome alignment pipelines based on LASTZ alignments running on a high-

performance computing cluster. G-Anchor successfully detects >96% of the total genome syntenic block length achievable by LASTZ.

The relative efficiency of G-Anchor is achieved by applying two major optimisation steps: a) the use of evolutionary highly conserved sequences (HCE) for whole-genome anchoring; b) "outsourcing" the computationally intensive step of defining the HCE to downloadable multiple whole-genome alignments prebuilt using traditional alignment methods. Once HCE are identified, they can be used for anchoring genomic sequences from a range of different genome combinations: this is because HCE are DNA sequences that are evolutionary conserved in a range of related and sometimes distant genomes. G-Anchor could be viewed as using HCE to quickly and accurately predict the alignment seeds that would be built by traditional aligners. Thus, the advantage of HCE anchors as compared to dynamically defined alignment seeds used in traditional aligners is that HCE anchors do not need to be built every time two genomes are compared. Instead G-Anchor is able to use a set of predefined HCE conserved across a wide range of vertebrate species and that are thus suitable for anchoring a range of genomes: i.e. the "Mammalian" set of HCE.

There is no need to have HCE databanks pre-defined for every possible ga-reference genome, instead a pre-processing script performs this task. Our results demonstrate that there is little penalty to be experienced when changing to a ga-reference that is distant from the reference genome used to create the original pairwise multiple whole-genome alignment, and that there was little effect of including or excluding the ga-target genome from the alignments used to identify HCE. Interestingly, the Cow-Yak set was capable of mapping 3 Mb more of yak sequence, distributed among small scaffolds, that was missed by the Cow+Yak set.

Probably the inclusion of Yak in the Cow+Yak alignment weakened the signal used to define the HCE that anchored these small scaffolds. These data demonstrate that G-Anchor is not only efficient in mapping scaffolds cross-species among two mammalian genome assemblies but is also flexible in using HCE sets defined with a different combination of genomes even when the ga-reference and ga-target were excluded from the process of HCE detection.

A very low number of inconsistently mapping scaffolds between G-Anchor and LASTZ alignments to cattle autosomes further proves the robustness of G-Anchor results. All the inconsistencies involve G-Anchor mapping a scaffold to a single ga-reference chromosome interval, while LASTZ aligns the same scaffold to more than one chromosome region (Fig. 6). One possible explanation for this is due to the higher resolution of LASTZ alignments, meaning that small intervals within scaffolds could be missed by the G-Anchor approach. This hypothesis is supported by our manual investigation of all inconsistencies. In all cases LASTZ and G-Anchor agree in chromosomal and regional assignment of the larger parts of the scaffolds. LASTZ, however, also assigns a smaller distinct part of the scaffold to another region of the same or different cattle autosome while G-Anchor fails to map this small fragment. On the other hand, it is possible that LASTZ aligns small parts of yak scaffolds to duplicated regions of cattle chromosomes that do not possess HCE anchors due to relaxed purifying selection in these regions. If this is true, G-Anchor could outperform LASTZ in mapping accuracy in such cases.

G-Anchor results prove our original hypothesis that HCE can be used as anchors for cross-species mapping for animal genomes. In mammals HCE constitute around 5-10% of the whole-genome sequence. This fraction is higher for closely related species (e.g. for ruminant species in our study) resulting in the ruminant HCE

dataset outperforming the 'Mammalian' HCE dataset in terms of the number of mapped scaffolds and comparative sequence coverage. In the avian genomes case the HCE constitute 9% of the whole-genome sequence. G-Anchor was able to successfully map 96% of the total Mallard's genome syntenic block length achieved by LASTZ, with only a slight increase in the number of inconsistencies. Based on this we expect that G-Anchor will work well for any group of species with high level of inter-species sequence conservation (e.g. mammals or birds) but likely be less efficient or even inefficient for comparison of related genomes with high level of sequence divergence (e.g. insects). Whole-genome duplications resulting in multiple chromosomes with similar sequence content, and a large fraction of repetitive elements, will likely make G-Anchor inefficient for anchoring many plant genomes.

In the case of more divergent sequences, the user should decrease the minimum similarity identity and the minimum alignment ratio, thus increasing the numbers of HCE anchors, and as a result the alignment coverage (Additional file 1, G-Anchor pipeline in Human – Mouse comparison). This has negligible effect on G-Anchor's running time. G-Anchor was found to be more efficient in anchoring larger scaffolds than smaller scaffolds due to a lower number of HCE anchors in the latter group. Therefore, the quantity of target genome assembly, for instance the scaffold lengths, could be another factor affecting G-Anchor efficiency.

The limiting factors mentioned above do not allow G-Anchor to be a substitute for whole-genome aligners but its ability to run on a workstation or laptop should allow G-Anchor to be widely used by small research groups and laboratories lacking access to HPC systems but still interested in whole-genome sequence comparison. Several additional optimization steps were applied to allow G-Anchor to run efficiently and produce the best possible results utilising a low amount of

computational resources. Splitting long HCE reduces the runtime, as well as providing a marginal increase in the number of mapped scaffolds. Most workstations and laptops now have multiple CPU cores which G-Anchor can utilize, decreasing the overall run time. HCE are highly conserved and non-repetitive sequences, thus allowing G-Anchor to use several optimization options available within BLAT that significantly reduce the time of the most computationally intensive and time-consuming G-Anchor step. Excluding highly repetitive DNA sequences from alignment seeding (-*ooc* option) and allowing to align only nearly identical sequences (-*fastMap* option) decreased the total time required for G-Anchor by a factor of 17, for the most computationally intensive HCE databank at cost of losing an insignificant number of mapped scaffolds.

In conclusion G-Anchor is an efficient cross-species genome anchoring pipeline suitable for execution on a personal computer. It allows for fast comparison of two species' genome assemblies that exhibit significant level of sequence conservation and are not highly repetitive or polypoid. G-Anchor could be used for fast identification of the regions of homologous synteny between genomes as well as for detection of scaffolds that might contain evolutionary breakpoints or assembly errors.

Availability and requirements

G-Anchor is portable and was designed to run on a MAC OSX or LINUX operating systems. The list of all command line options for G-Anchor is fully described in the user manual available at GitHub (https://github.com/vasilislenis/G-Anchor). An archival copy of the code, test input data and HCE databanks are available via the *GigaScience* repository GigaDB [28].

- **Project name:** G-Anchor
- **Project home page:** https://github.com/vasilislenis/G-Anchor
- **Operating systems:** MAC OSX, LINUX
- **Programming languages:** Bash, Perl
- **Other requirements:** none
- **License:** The MIT license (MIT)
- **RRID:** SCR_016046
- **Any restriction to use by non-academics:** none

Competing interests

The authors declare that they have no competing interests.

Authors' contributions

VPL implemented the software. DML and MTS conceived the project and contributed equally to the project. All authors structured the draft, provided final editing, and approved the final manuscript.

Acknowledgements

The authors thank HPC Wales and Fujitsu for providing the resources and support. We also thank the team at UCSC for the tools, genome sets and timely clarifications. This work was supported in part by the Biotechnology and Biological Sciences Research Council grant BB/J010170/1 to DML.

References

[1] P. Avdeyev, S. Jiang, S. Aganezov, F. Hu, and M. A. Alekseyev, "Reconstruction of ancestral genomes in presence of gene gain and loss.," *bioRxiv*, p. 40196, Feb. 2016.

[2] M. Rocchi, N. Archidiacono, and R. Stanyon, "Ancestral genomes reconstruction: An integrated, multi-disciplinary approach is needed," *Genome Res.*, vol. 16, no. 12, pp. 1441–1444, Dec. 2006.

[3] G. Wächtershäuser, "Towards a Reconstruction of Ancestral Genomes by Gene Cluster Alignment," *Syst. Appl. Microbiol.*, vol. 21, no. 4, pp. 473–477, Dec. 1998.

[4] R. Ekblom and J. B. W. Wolf, "A field guide to whole-genome sequencing, assembly and annotation," *Evol. Appl.*, vol. 7, no. 9, pp. 1026–1042, Nov. 2014.

[5] Z. D. Stephens *et al.*, "Big Data: Astronomical or Genomical?," *PLOS Biol*, vol. 13, no. 7, p. e1002195, Jul. 2015.

[6] D. Tagu, J. K. Colbourne, and N. Nègre, "Genomic data integration for ecological and evolutionary traits in non-model organisms," *BMC Genomics*, vol. 15, no. 1, Jul. 2014.

[7] K.-P. Koepfli, B. Paten, Genome 10K Community of Scientists, and S. J. O'Brien, "The Genome 10K Project: a way forward," *Annu. Rev. Anim. Biosci.*, vol. 3, pp. 57–111, 2015.

[8] L. Fontanesi *et al.*, "LaGomiCs—Lagomorph Genomics Consortium: An International Collaborative Effort for Sequencing the Genomes of an Entire Mammalian Order," *J. Hered.*, vol. 107, no. 4, pp. 295–308, Jul. 2016.

[9] P. A. Kitts *et al.*, "Assembly: a resource for assembled genomes at NCBI," *Nucleic Acids Res.*, vol. 44, no. D1, pp. D73–D80, Jan. 2016.

[10] F. Cunningham, M. R. Amode, D. Barrell, K. Beal, K. Billis, and S. Brent, "Ensembl 2015," *Nucleic Acids Res*, vol. 43, 2015.

[11] R. M. Kuhn, D. Haussler, and W. J. Kent, "The UCSC genome browser and associated tools," *Brief. Bioinform.*, vol. 14, no. 2, pp. 144–161, Mar. 2013.

[12] S. Santini, J. L. Boore, and A. Meyer, "Evolutionary Conservation of Regulatory Elements in Vertebrate Hox Gene Clusters," *Genome Res.*, vol. 13, no. 6a, pp. 1111–1122, Jun. 2003.

[13] A. Siepel *et al.*, "Evolutionarily conserved elements in vertebrate, insect, worm, and yeast genomes," *Genome Res.*, vol. 15, no. 8, pp. 1034–1050, Aug. 2005.

[14] D. Villar *et al.*, "Enhancer Evolution across 20 Mammalian Species," *Cell*, vol. 160, no. 3, pp. 554–566, Jan. 2015.

[15] G. Zhang *et al.*, "Comparative genomics reveals insights into avian genome evolution and adaptation," *Science*, vol. 346, no. 6215, pp. 1311–1320, Dec. 2014.

[16] G. Zhang, B. Li, C. Li, M. T. P. Gilbert, E. D. Jarvis, and J. Wang, "Comparative genomic data of the Avian Phylogenomics Project," *GigaScience*, vol. 3, p. 26, 2014.

[17] A. L. Delcher, S. Kasif, R. D. Fleischmann, J. Peterson, O. White, and S. L. Salzberg, “Alignment of whole genomes,” *Nucleic Acids Res.*, vol. 27, no. 11, pp. 2369–2376, Jan. 1999.
[18] R. S. Harris, “Improved pairwise alignment of genomic DNA.” The Pennsylvania State University., 2007.
[19] W. J. Kent, R. Baertsch, A. Hinrichs, W. Miller, and D. Haussler, “Evolution’s cauldron: Duplication, deletion, and rearrangement in the mouse and human genomes,” *Proc. Natl. Acad. Sci.*, vol. 100, no. 20, pp. 11484–11489, Sep. 2003.
[20] M. Blanchette *et al.*, “Aligning Multiple Genomic Sequences With the Threaded Blockset Aligner,” *Genome Res.*, vol. 14, no. 4, pp. 708–715, Apr. 2004.
[21] A. R. Quinlan and I. M. Hall, “BEDTools: a flexible suite of utilities for comparing genomic features,” *Bioinformatics*, vol. 26, no. 6, pp. 841–842, Mar. 2010.
[22] J. Damas *et al.*, “Upgrading short read animal genome assemblies to chromosome level using comparative genomics and a universal probe set,” *Genome Res.*, p. gr.213660.116, Nov. 2016.
[23] W. J. Kent, “BLAT—The BLAST-Like Alignment Tool,” *Genome Res.*, vol. 12, no. 4, pp. 656–664, Apr. 2002.
[24] H. Li, “Aligning sequence reads, clone sequences and assembly contigs with BWA-MEM,” *ArXiv13033997 Q-Bio*, Mar. 2013.
[25] H. Li, “Minimap and miniasm: fast mapping and de novo assembly for noisy long sequences,” *Bioinformatics*, vol. 32, no. 14, pp. 2103–2110, Jul. 2016.
[26] N. Stojanovic *et al.*, “Comparison of five methods for finding conserved sequences in multiple alignments of gene regulatory regions,” *Nucleic Acids Res.*, vol. 27, no. 19, pp. 3899–3910, Oct. 1999.
[27] “icebert/pblat,” *GitHub*. [Online]. Available: https://github.com/icebert/pblat. [Accessed: 06-Apr-2017].
[28] Panagiotis V, Lenis E, Swain M, Larkin DM. Supporting data for "G-Anchor: a novel approach for whole-genome comparative mapping utilising evolutionary conserved DNA sequences". *GigaScience* database 2018. http://dx.doi.org/10.5524/100415

Tables

Table 1. HCE datasets statistics.

	Cattle ga-reference				Human ga-reference				
	Cow+Yak		Cow-Yak		Hum+Rum		Hum+Mam		
	Mapped*	Uniquely mapped**	Mapped	Uniquely mapped	Mapped	Uniquely mapped	Mapped	Uniquely mapped	Split & uniquely mapped
Total number	851161	850947	853562	853348	793064	275924	2139902	360322	395919
Total length (Mb)	416	416	431	431	146	45	289	54	61
Max HCE length (bp)	12482	12482	13715	13715	17204	17143	17204	17163	505
Min HCE length (bp)	40	40	40	40	40	40	40	40	40
Median (bp)	369	369	382	382	134	134	96	119	129
Genome fraction	16.38%	16.38%	16.95%	16.95%	5.7%	1.78%	11.4%	2.1%	2.4%

*Only HCE >40 bp were included. **HCE databank mapping to unique positions in the cattle autosomes. The length of some HCE can be slightly increased after the mapping due to gap presence.

Table 2. Aligned and anchored scaffold statistics.

	LASTZ-based alignments	Cow+Yak	Cow-Yak	Hum+Rum	Hum+Mam	Hum+Mam (split)
Number of anchored scaffolds*	3550 (100%)	3144 (89%)	3160 (89%)	2923 (82%)	3012 (85%)	3015 (85%)
Inconsistencies**	N/A	15	16	15	12	12
Total length of anchored scaffolds (Mb)	2535 (100%)	2458 (97%)	2454 (96.8%)	2434 (96%)	2,445 (96.4%)	2445 (96.4%)
N50	1567874	1580499	1584378	1539131	1539025	1539025
Median (bp)	368395	443232	433945	501467	472660	472660

*Scaffolds included >= 10 Kb. **Number of scaffolds that G-Anchor mapped to a single chromosome and LASTZ partially to more than one chromosomes.

Table 3. Additional scaffolds mapped using one of the two cattle-based HCE sets and their statistics.

	Cow+Yak	Cow-Yak
No. additional scaffolds*	29	45
Total length (Mb)	7.5	2.8
N50 (bp)	1113607	102029
Max. scaffold length (bp)	1522230	422417
Min. scaffold length (bp)	10407	10074
Median (bp)	63483	29826

*Additional scaffolds were mapped using one of the two cattle-based HCE sets but not when another set was used.

Table 4. Effect on mapping results when using BLAT parameters on Cow-Yak dataset.

	Mapped scaffolds	Coverage	Time on a singe core (min)	Time on 4 cores (min)
None parameters	3160	96.80%	7200	2880
All parameters	3149	96.51%	420	194
fastMap	3149	96.51%	780	323
ooc	3160	96.80%	1980	437

Table 5. Effect on mapping results when using BLAT parameters on Hum+Mam dataset.

	Mapped scaffolds	Coverage	Time on a singe core (min)	Times on 4 cores (min)
None parameters	3012	93.76%	132	95
All parameters	3001	93.16%	91	85
fastMap	3001	93.16%	120	89
ooc	3012	93.76%	123	91

Table 6. Total preprocessing time for changing the databank reference genome with the Mammalian HCE data set (from human to cattle).

	Single core		Four cores	
	No. of HCE*	Preprocessing time (min)	No. of HCE*	Preprocessing time (min)
None parameters	360322	287.46	360322	100
All parameters	186655	122.37	186655	47.37
fastMap	186655	140.47	186655	55.34
ooc	360322	188.13	360322	52.25

* Total number of HCE that are that are mapped to the new ga-reference genome (cattle) and can be used by G-Anchor for the mapping of yak genome.

Figures

Figure 1. Genome alignment and HCE prediction pipeline.

Figure 2. G-Anchor pipeline workflow. Numbered boxes identify different stages of the pipeline workflow.

Figure 3. Lengths' of potential anchors. HCE >= 40 bp and aligned uniquely in the cattle ga-reference. (a). The HCE sets built from the cattle genome-based alignments (b). The HCE sets built from the human genome-based alignments.

Figure 4. Fraction of the Hum+Mam databank that aligned to unique ga-reference chromosome and target scaffold positions before and after splitting.

Figure 5. Number of yak scaffolds mapped to each cattle autosome using four HCE databanks and the whole-genome LASTZ alignments.

Figure 6. Schematic presentation of the discrepancies observed between mappings of scaffolds by LASTZ and G-Anchor.

Figure 7. G-Anchor run times per ga-reference chromosome. G-Anchor was used to map yak scaffolds against the cattle genome with Cow-Yak HCE set: a) no alignment optimization parameters using one and four cores; b) applying –ooc, -fastMap and both of these parameters using one core; c) applying –ooc, -fastMap and both of these parameters using four cores.

Additional files

Additional file 1

Supp_material:

1. HCE databank construction (parameters)

Table 1: Alignment, chains & nets construction and HCE prediction parameters.

2. Comparison of intersecting fraction and mapped genome coverage

Figure 1: Intervals overlapping ratio.

3. Mapping Inconsistencies

Figure 2: UCSC genome browser snapshot of cattle chromosome 18 and nets of yak scaffolds 473 and 776.

4. Avian genomes: HCE databank and G-Anchor's results

Table 2: HCEs from Avian databank. General statistics.

Table 3: Statistics and coverage of the Mallard's genome anchoring.

5. G-Anchor pipeline in Human – Mouse comparison

Table 4: Mapping coverage status by using different values in HCE alignment and filtering

Figure 3: HCE anchors in Human-Mouse comparison.

Figure 5: HCE that were aligned and filtered using different criteria (in terms of coverage).

6. G-Anchor and Minimap: Times comparison

Figure 6: G-Anchor and Minimap running times.

Additional file 2

Cow+Yak

Comparison of G-Anchor's and LASTZ based nets results in Cow+Yak dataset

Additional file 3

Cow-Yak

Comparison of G-Anchor's and LASTZ based nets results in Cow-Yak dataset

Additional file 4

Hum+Rum

Comparison of G-Anchor's and LASTZ based nets results in Hum+Rum dataset

Additional file 5

Hum+Mam

Comparison of G-Anchor's and LASTZ based nets results in Hum+Mam dataset

Additional file 6

Hum+Mam-250

Comparison of G-Anchor's and LASTZ based nets results in Hum+Mam (split in 250bp) dataset

Figure 1

Click here to download Figure Fig1.tiff

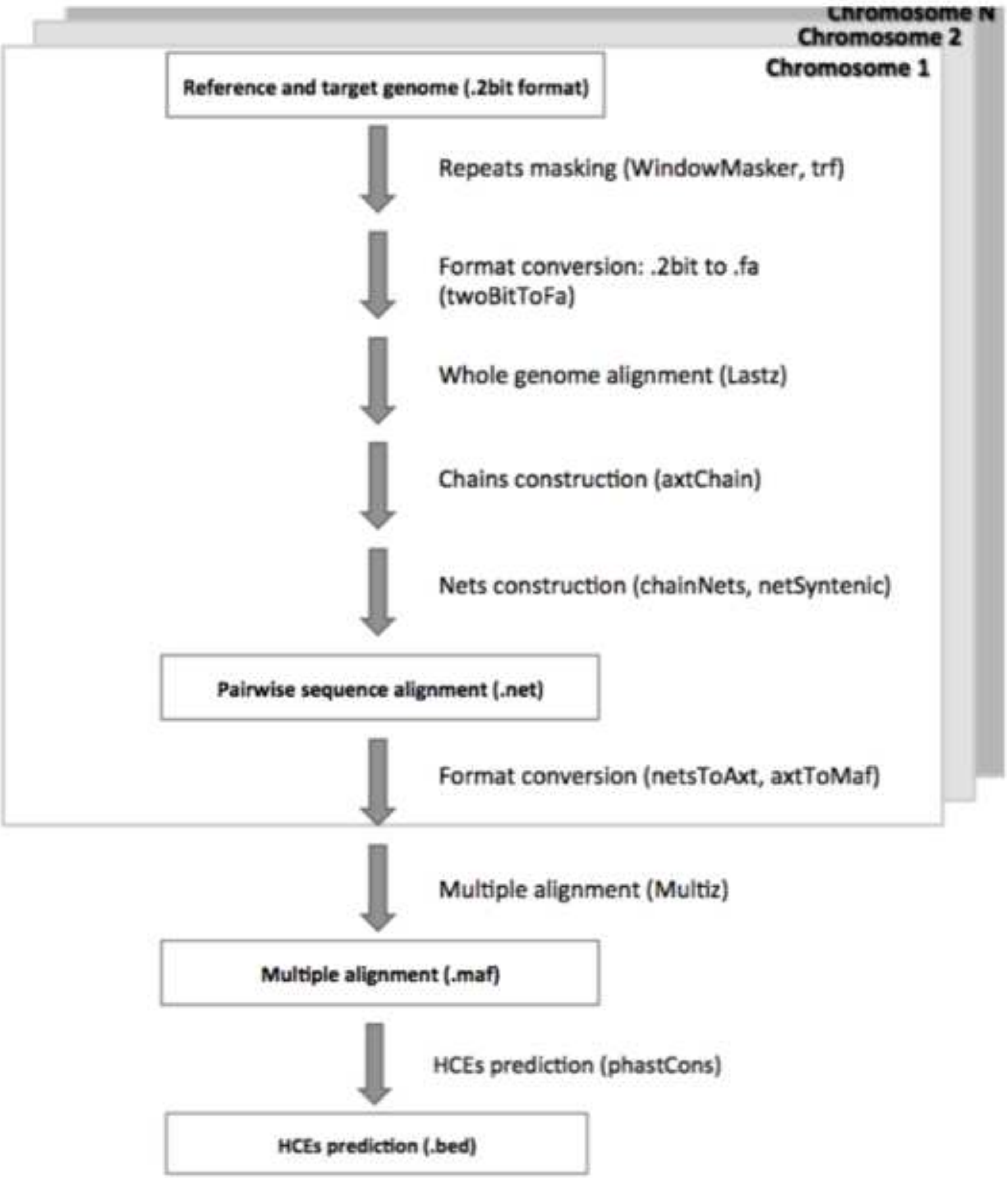

Figure 2

Click here to download Figure Fig2.jpg

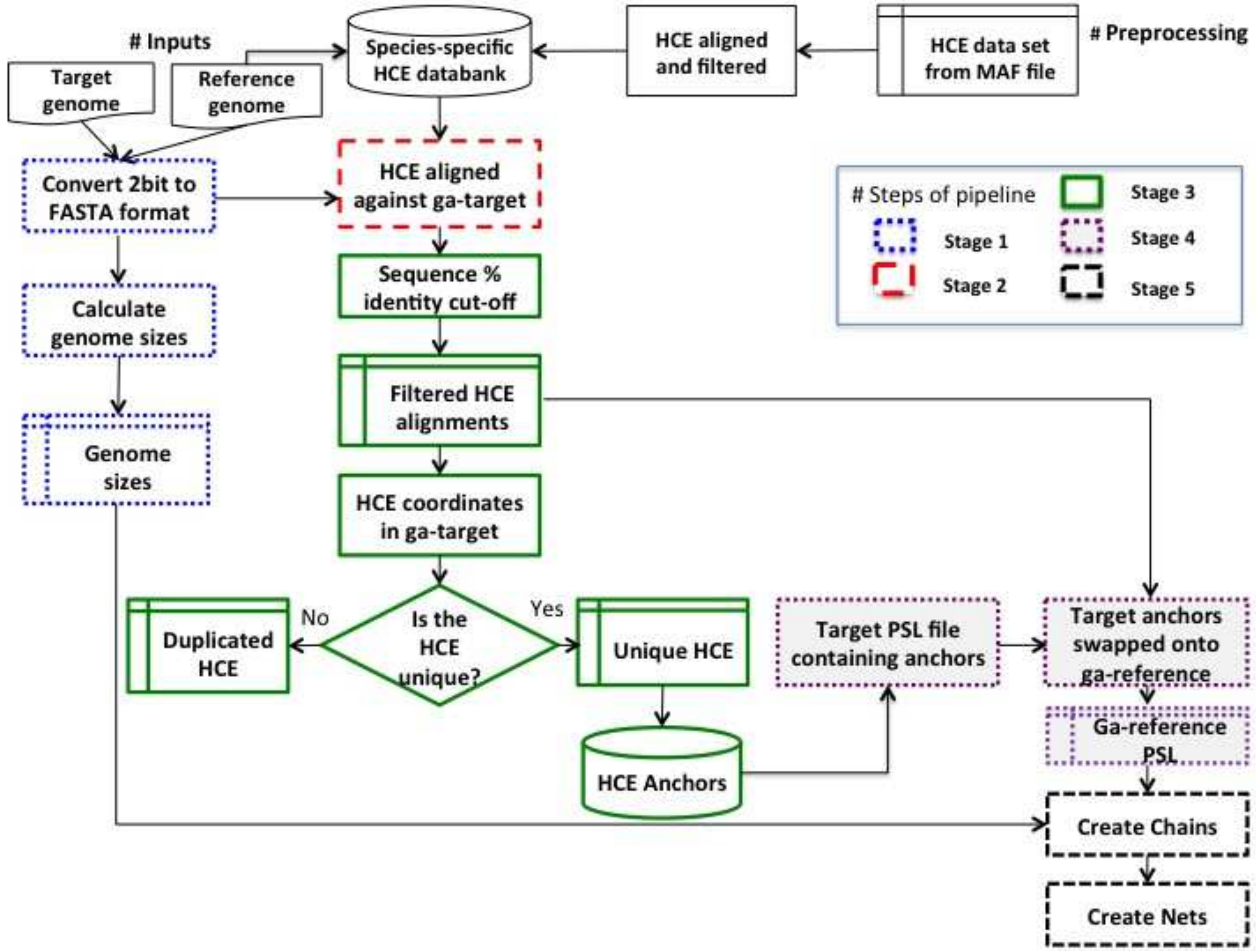

Figure 3

Click here to download Figure Fig3.tiff

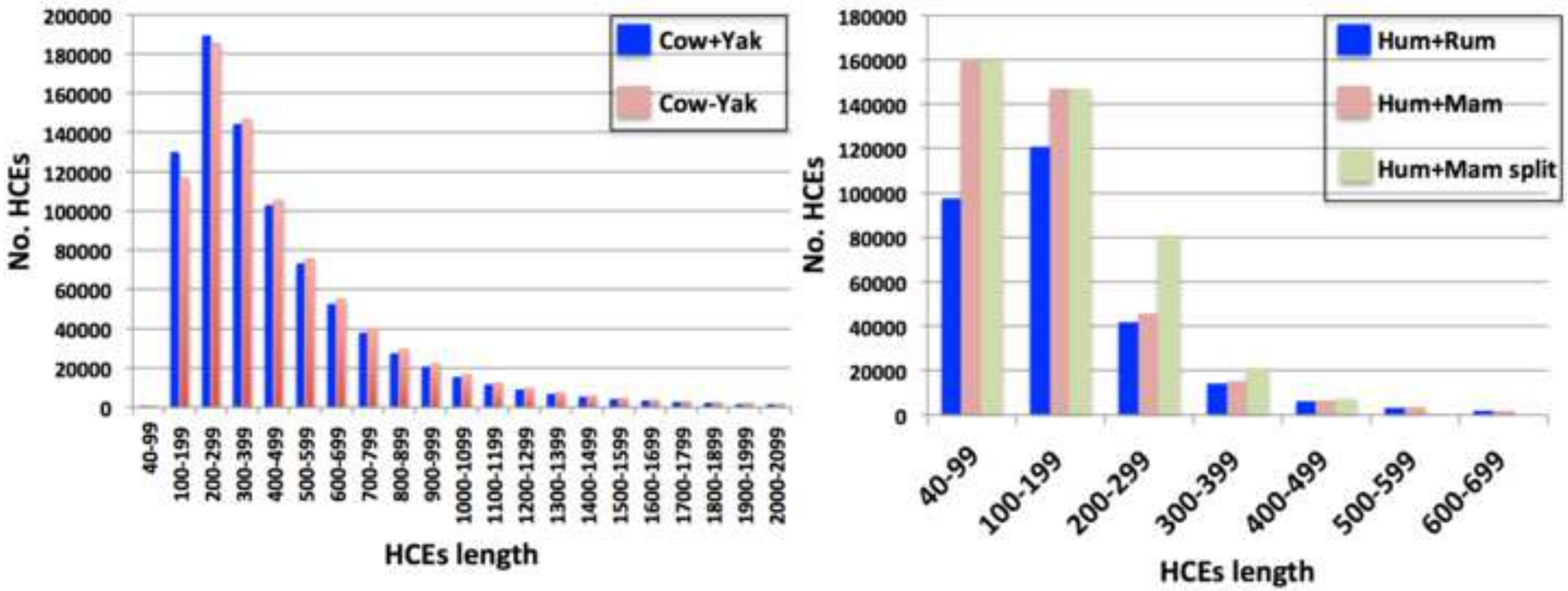

Figure 4

Click here to download Figure Fig4.tiff

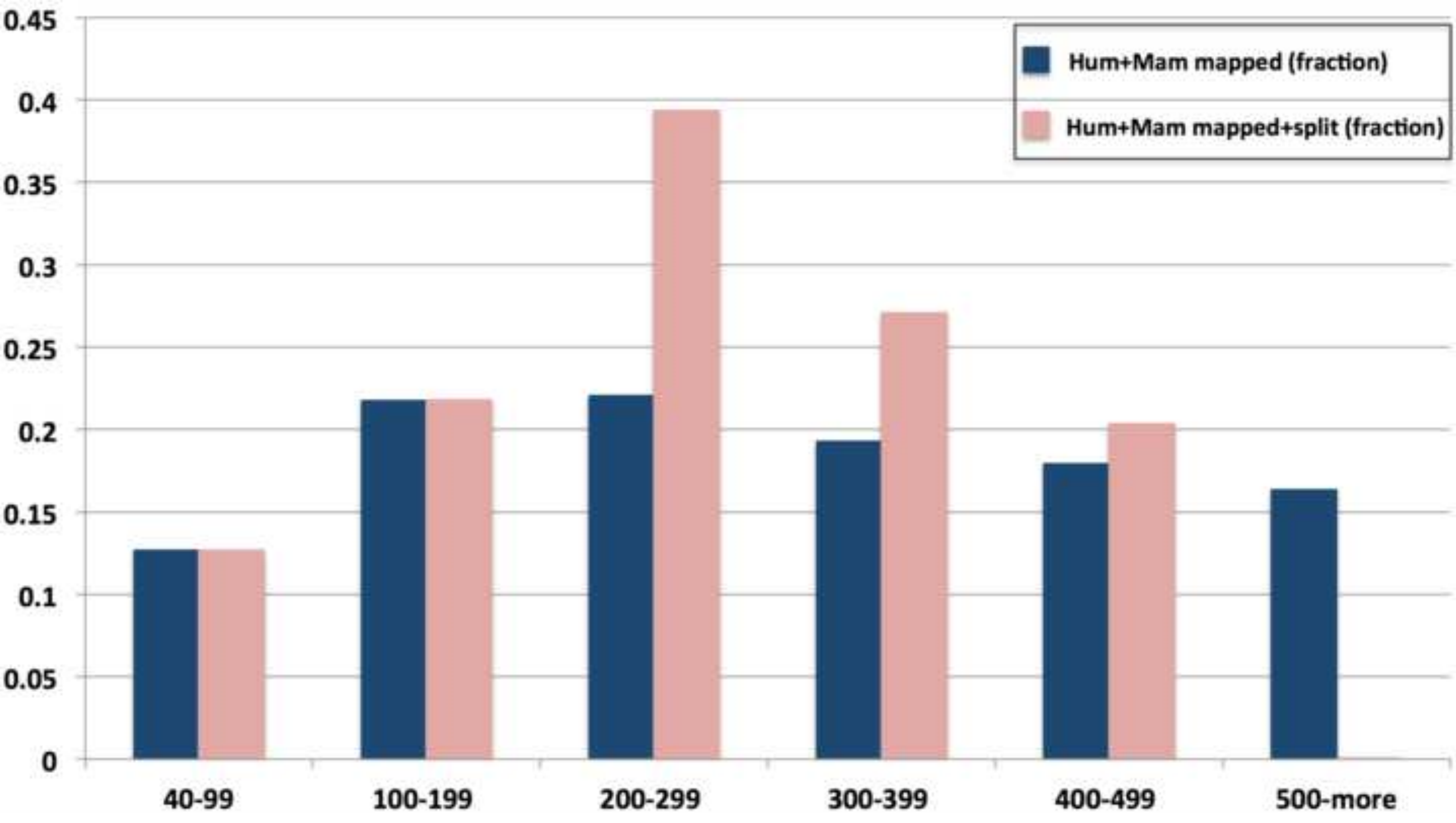

Figure 5

Click here to download Figure Fig5.tiff

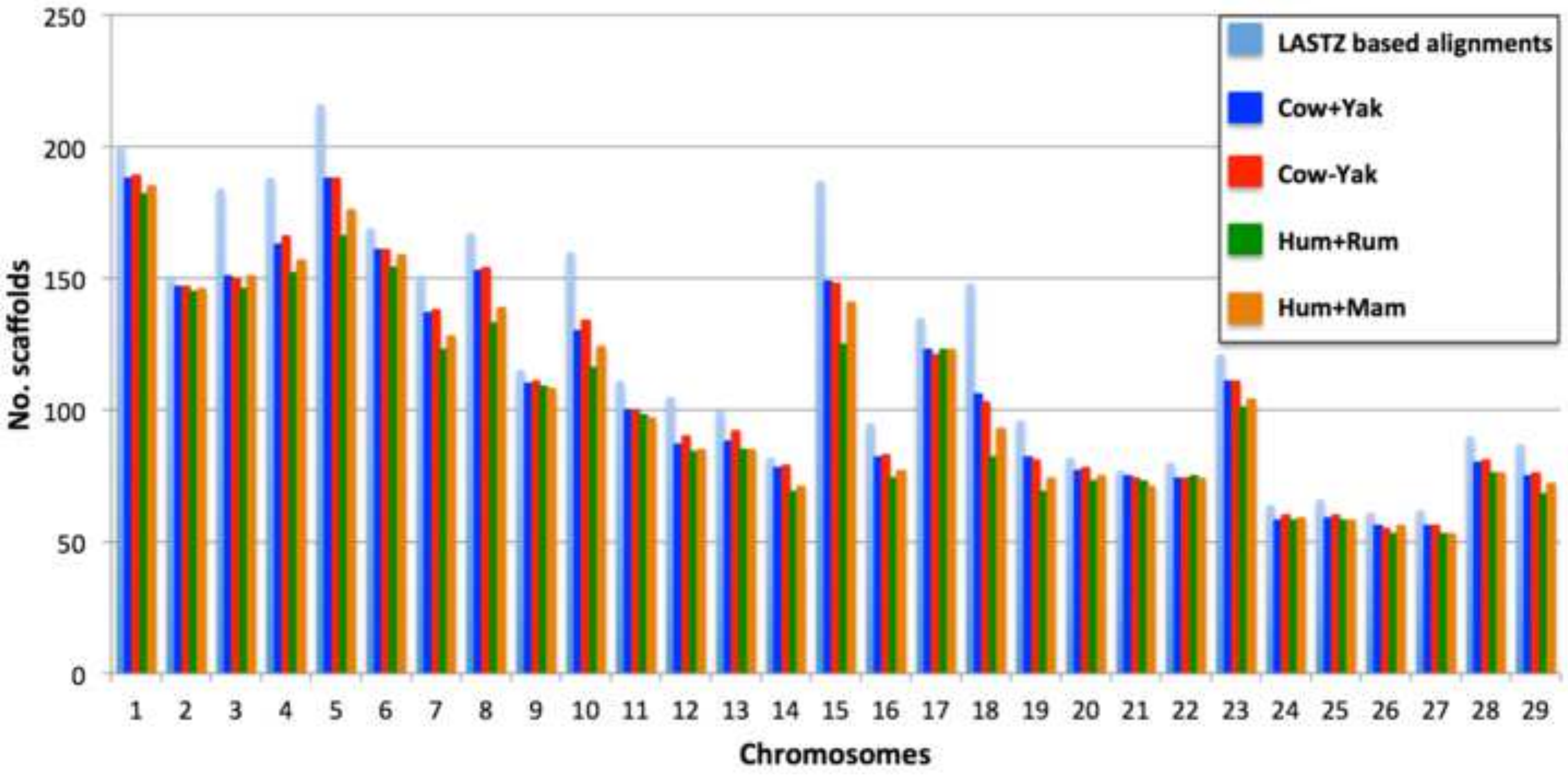

Figure 6

Click here to download Figure Fig6.tiff

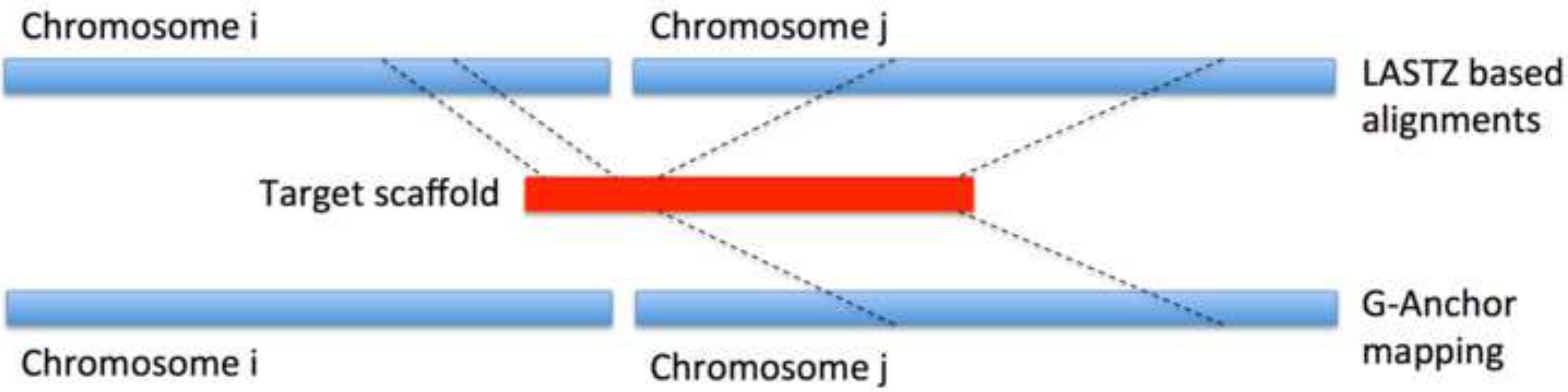

Figure 7 Click here to download Figure Fig7.tiff

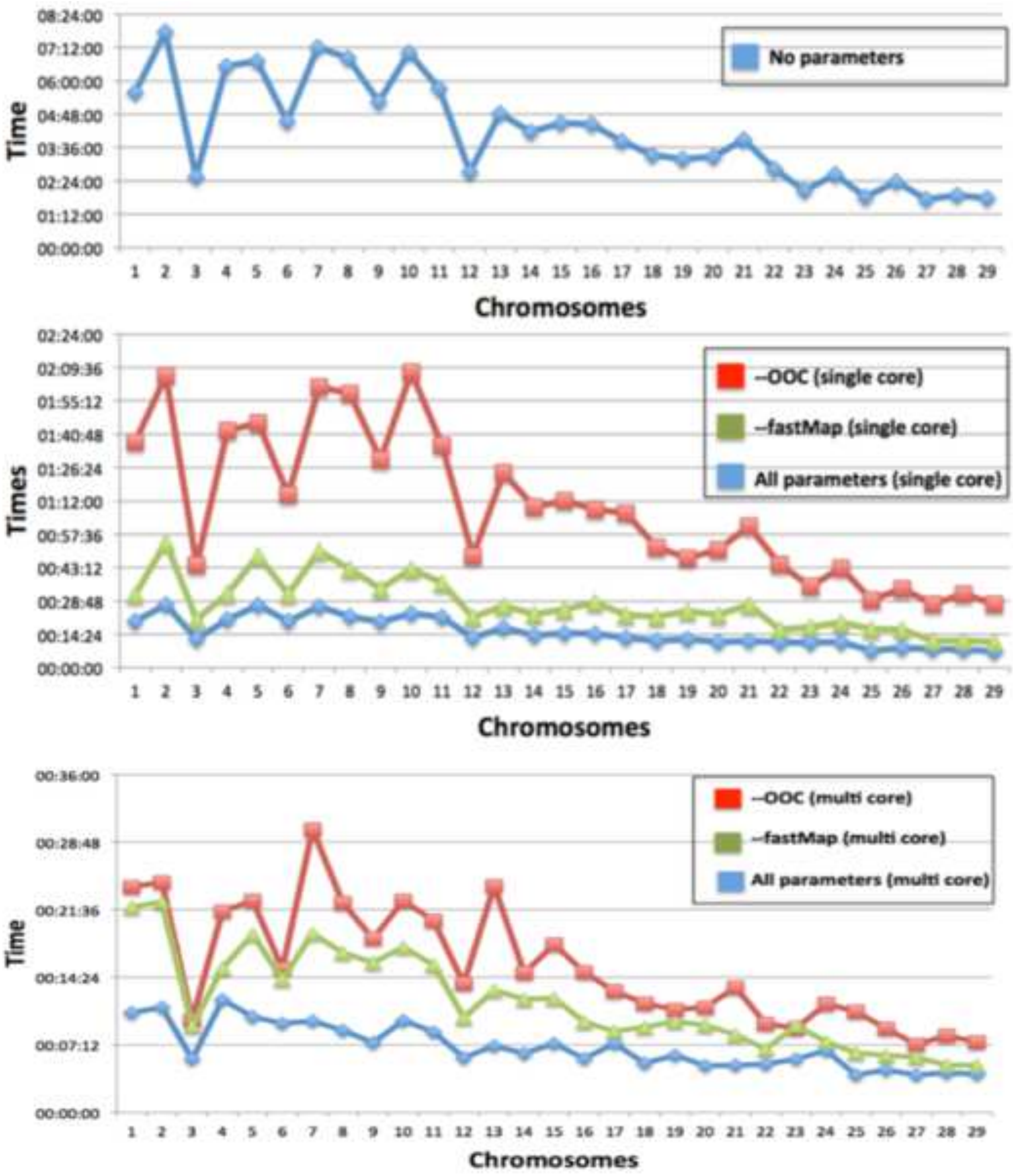

Additional file 1

Click here to access/download
Supplementary Material
Supp_material.docx

Additional file 2

Click here to access/download
Supplementary Material
Cow+Yak.xls

Additional file 3

Click here to access/download
Supplementary Material
Cow-Yak.xls

Additional file 4

Click here to access/download
Supplementary Material
Hum+Rum.xls

Additional file 5

Click here to access/download
Supplementary Material
Hum+Mam.xls

Additional file 6

Click here to access/download

Supplementary Material

Hum+Mam-250.xls

Click here to download Personal Cover Cover letter Feb2018.docx

We thank for the reviewers for their further feedback. Our response to their suggestions is as follows.

Reviewer 1:

To more easily compare the run-time metrics of LASTZ, MUMmer, and G-Anchor, please report how long a whole genome alignment of the yak and cattle genomes takes using MUMmer 4.0. Please report in the same paragraph as LASTZ and MUMmer the total run-time of all stages of the G-Anchor pipeline for mapping the Yak genome to the Cow genome. Please report the multi-threaded wall-clock run-times of MUMmer and G-Anchor, noting that LASTZ is not multithreaded.

We have added this material between lines 505 and 544. Briefly:

- We give the time for LASTZ noting that is not multi-threaded (5 days, 4 hours).
- We report that MUMmer 4.0 takes 48 hours with a single core and 12 hours with 4 cores, and requires 43 GB RAM in both cases.
- G-Anchor requires 7 hours with a single core and 3 hours 15 minutes for 4 cores with 4.5 GB RAM. For the single core case, the times for the five stages are 4, 12, 11, 353, and 43 minutes respectively. Aligning the HCE with Blat takes the longest time: this was reduced from 353 (1 core) to 124 minutes (4 cores).

Reviewer 2:

Minor comments: 1. Combining math and (pseudo)code (e.g., Page 19 equation), while may serve the purpose of conveying the notion, may be written clearer using a standard pure math or pure algorithm notation.

We have added a standard pure math notation of the equation, between lines 436 to 444, as requested.

Editor:

In addition, please register any new software application in the SciCrunch.org database to receive a RRID (Research Resource Identification Initiative ID) number, and include this in your manuscript. This will facilitate tracking, reproducibility and re-use of your tool.

We have registered the software and added the RRID number (SCR_016046) at line 666.
