# Supplementary material for: G-Anchor: a novel approach for whole-genome comparative mapping utilizing evolutionary conserved DNA sequences
Source: Gigascience. 2018 Apr 3;7(5):giy017. doi: 10.1093/gigascience/giy017 (PMC5961299; doi:10.1093/gigascience/giy017)
Supplement: Additional Files [file giy017_supp.zip › Supp_material.docx]

**HCE dataset construction (parameters)**

**Table 1: Alignment, chain and net construction parameters**

|  | Cow+Yak | Cow-Yak | Hum+Rum | Hum+Mam |
| --- | --- | --- | --- | --- |
| LASTZ | E=30, H=2000, K=3000, L=2200, O=400, default substitution matrix | | | |
| axtChain | -verbose=0 -linearGap=medium -minScore=3000 | | | |
| MULTIZ | Default parameters | | | |
| phastCons | expected-length: 45  target-coverage: 0.45  rho: 0.31 | expected-length: 45 target-coverage: 0.463  rho: 0.31 | expected-length: 45 target-coverage: 0.5 rho: 0.32 | \| Primates:  expected-length: 45 target-coverage: 0.5  rho: 0.32 \| \| --- \| \| Rodents:  expected-length: 45 target-coverage: 0.3  rho: 0.31 \| \| Carnivores:  expected-length: 45  target-coverage: 0.2  rho: 0.30 \| |

**Comparison of intersecting fraction and mapped genome coverage**


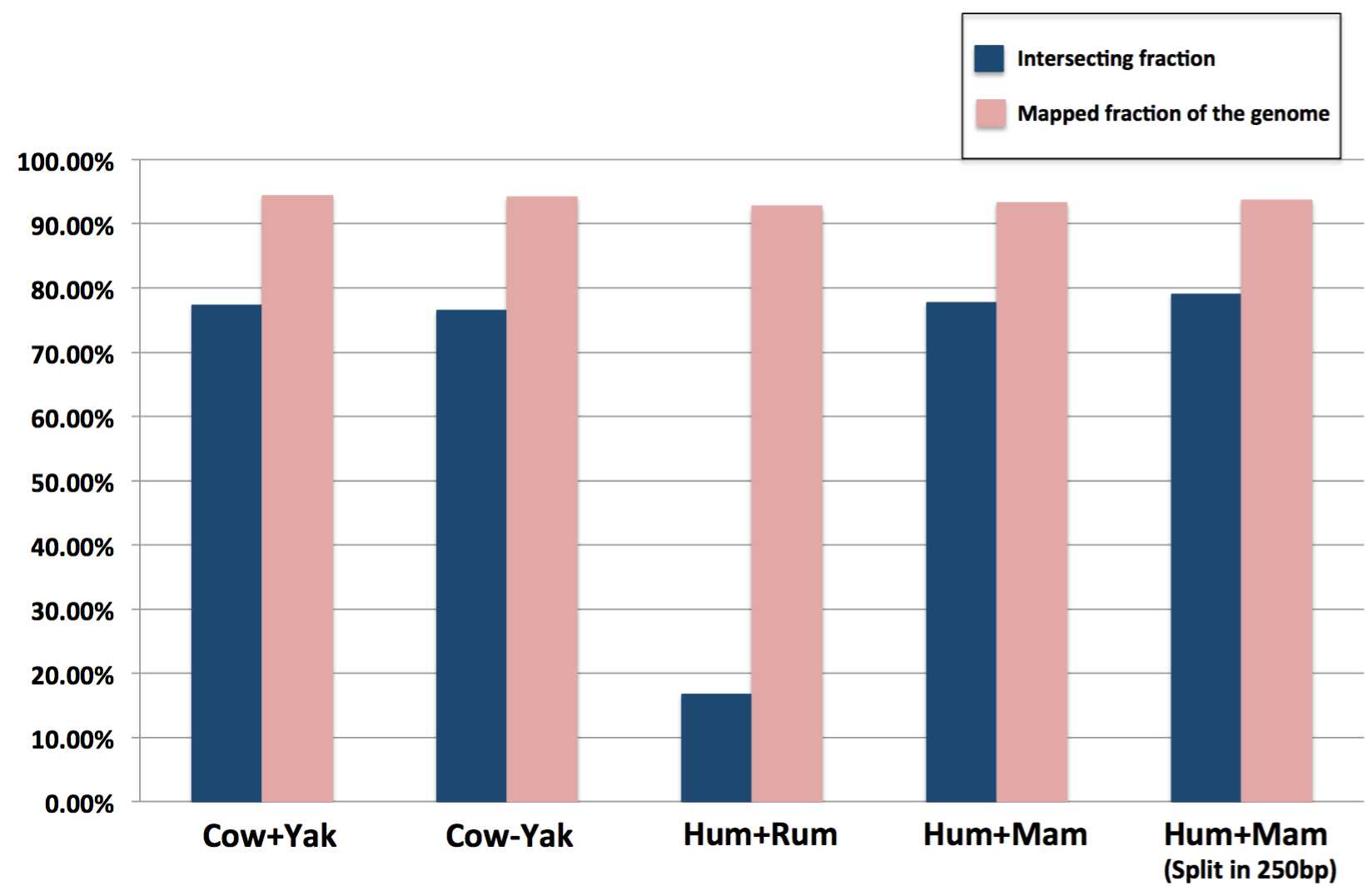


Figure 1: Intersecting fraction. The intersecting fraction was calculated by dividing the common aligned bases in nets for the both of the two methods (ours pipeline and LASTZ based alignment method) with the total number of bases in the LASTZ based alignment nets. Even if the overlapping ratio was decreased when the human based ruminants’ dataset was used for the alignments, the genome fraction remained at the same level.

**Mapping Inconsistencies**


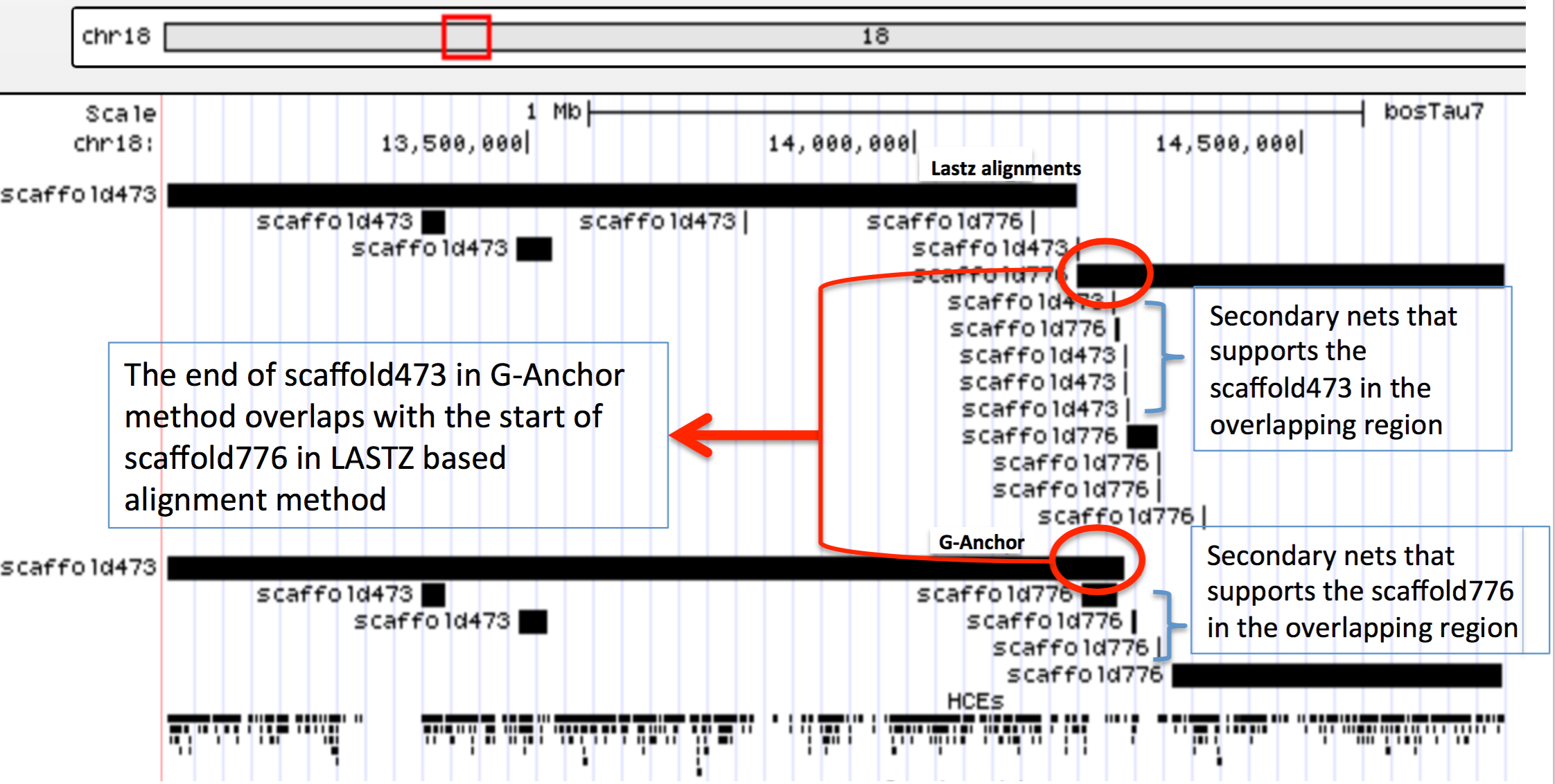


Figure 2: A screenshot of cattle chromosome 18:13,640,415-15,148,055 bps, showing nets of the yak scaffolds 473 and 776. The “Lastz alignments” tracks show the LASTZ based alignments. The “G-Anchor” track shows the G-Anchor mapping results by using the Cow-Yak dataset.

**Avian genomes: HCE data set and G-Anchor’s results**

The avian HCE data set was constructed by applying the methodology that is described in Section 2 (“Data preparation and processing”). The 20 avian genomes that participated in the HCE data set are: Ostrich *(Struthio camelus australis)*, Mallard *(Anas Platyrhynchos)*, Turkey *(Meleagris gallopavo)*, Rock pigeon *(Columba livia)*, Common cuckoo *(Cuculus canorus canorus)*, Chimney swift *(Chaetura pelagica)*, Anna’s hummingbird *(Calypte anna)*, Hoatzin *(Opisthocomus hoazin)*, Killdeer *(Charadrius vociferus vociferous)*, Emperor penguin *(Aptenodytes forsteri)*, Adelie penguin *(Pygoscelis adeliae)*, Crested ibis *(Nipponia Nippon)*, Little egret *(Egretta garzetta)*, Peregrine falcon *(Falco peregrinus)*, Budgerigar *(Melopsittacus undulates)*, Golden collared manakin *(Manacus vitellinus)*, American crow *(Corvus brachyrhynchos)*, Zebra finch *(Taeniopygia guttata)*, Medium ground finch *(Geospiza fortis)* and Downy woodpecker *(Picoides pubescens)*. The reference genome that it was used is chicken (*Gallus gallus*).

Table 2: HCEs from Chi-Mal data set. General statistics.

Less than a half of the predicted HCE (Mapped) managed to map on the reference genome in unique positions (Uniquely mapped). Due to the large diversity of the bird genomes there were predicted elements that are quite small (min length 1 bp) that could be mapped on several different places on the reference genome. The unique elements that remained after filtering are larger than 30 bp and 59 bp median lengths.

|  | Chicken reference | |
| --- | --- | --- |
|  | Chi-Mal | |
|  | Mapped | Uniquely mapped |
| Total number | 2100664 | 951501 |
| Total length (Mb) | 103 | 84 |
| Max HCE length (bp) | 3348 | 3348 |
| Min HCE length (bp) | 1 | 30 |
| Median (bp) | 27 | 59 |
| Genome fraction | 11.3% | 9.1% |

Table 3: Statistics and coverage of the Mallard’s genome anchoring.

G-Anchor managed to map the majority of the large scaffolds (Total length) that covers more than 96% of the scaffolds that were aligned by LASTZ. It also placed a number of scaffolds in a different chromosome (inconsistencies) based on strong conservation evidences.

|  | LASTZ-based alignments | Chi-Mal |
| --- | --- | --- |
| Number of anchored scaffold* | 1801 (100%) | 1580 (88%) |
| Inconsistencies** | N/A | 25 |
| Total length of anchored scaffolds (Mb) | 1032 (100%) | 991 (96%) |
| N50 | 1305515 | 1317219 |
| Median (bp) | 283269 | 320705 |

**G-Anchor pipeline in Human – Mouse comparison**

Table 4: Mapping coverage status by using different values in HCE mapping and filtering. *–minIdentity* parameter represents the down threshold of the similarity identity in the mapping stage. On the other hand, *–minAli* represents the filtering threshold in order to keep the best HCE alignment. By relaxing these values when G-Anchor is used with more distant related species like human and mouse, an increasing in the number of the HCE anchors is noticed which led to an increasing in the mapping coverage.

| Human-Mouse | Coverage | HCE Anchors |
| --- | --- | --- |
| Default | 35.17% | 3.32% |
| -minIdentity=90  -minAli=0.90 | 45.71% | 8.77% |
| -minIdentity=85  -minAli=0.85 | 82.73% | 15.97% |
| -minIdentity=80  -minAli=0.80 | 88.90% | 18.10% |
| -minIdentity=75  -minAli=0.75 | 88.61% | 18.35% |


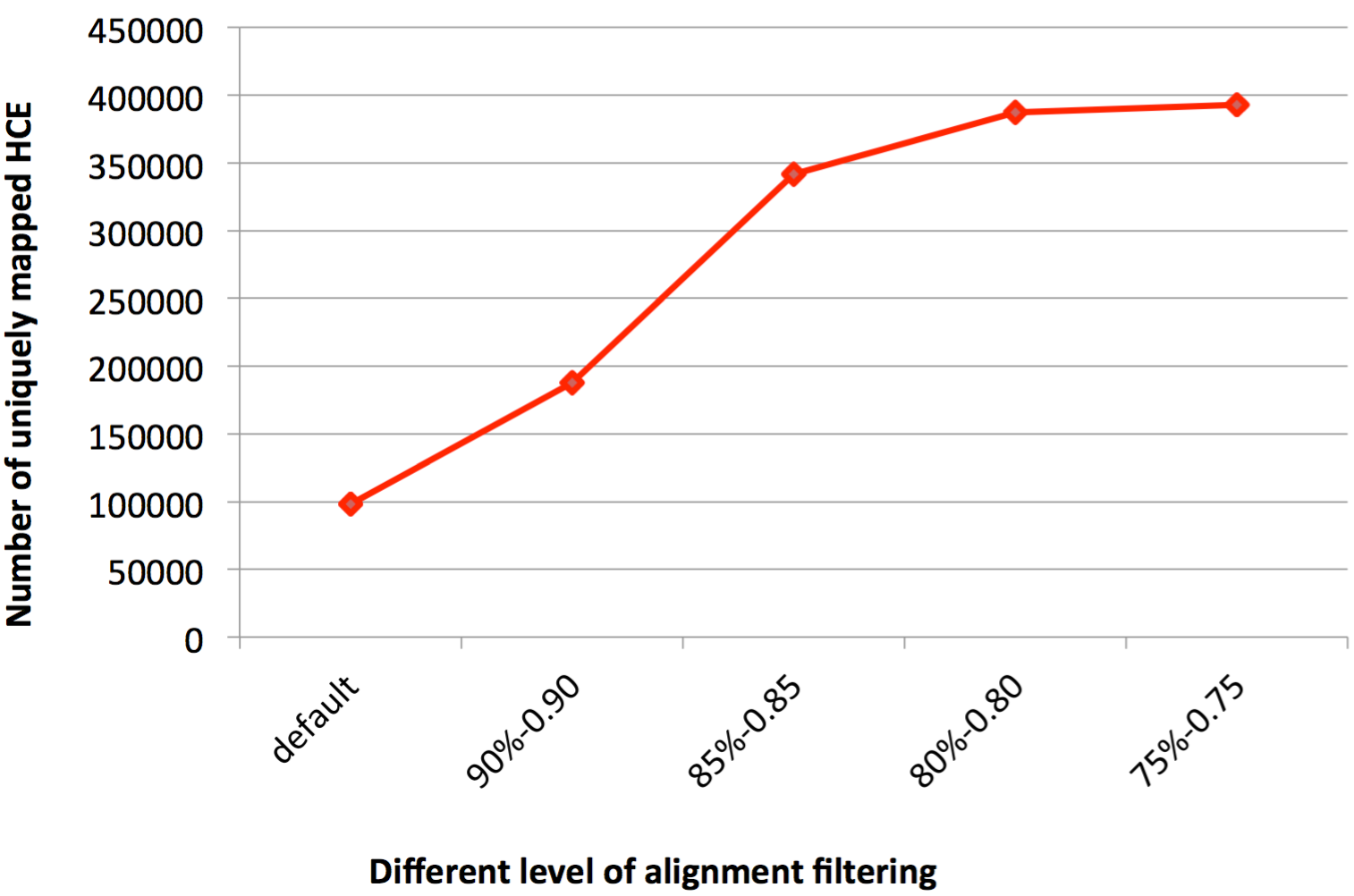


Figure 3: HCE anchors in Human-Mouse comparison. By using more relaxed criteria in the stage 2 (-minIdentity parameter (%)) and stage 3 (-minAli parameter (decimal)) G-Anchor managed to increase the number of the HCE anchors (uniquely mapped HCE). As a consequence, the mapping coverage was increased as well.


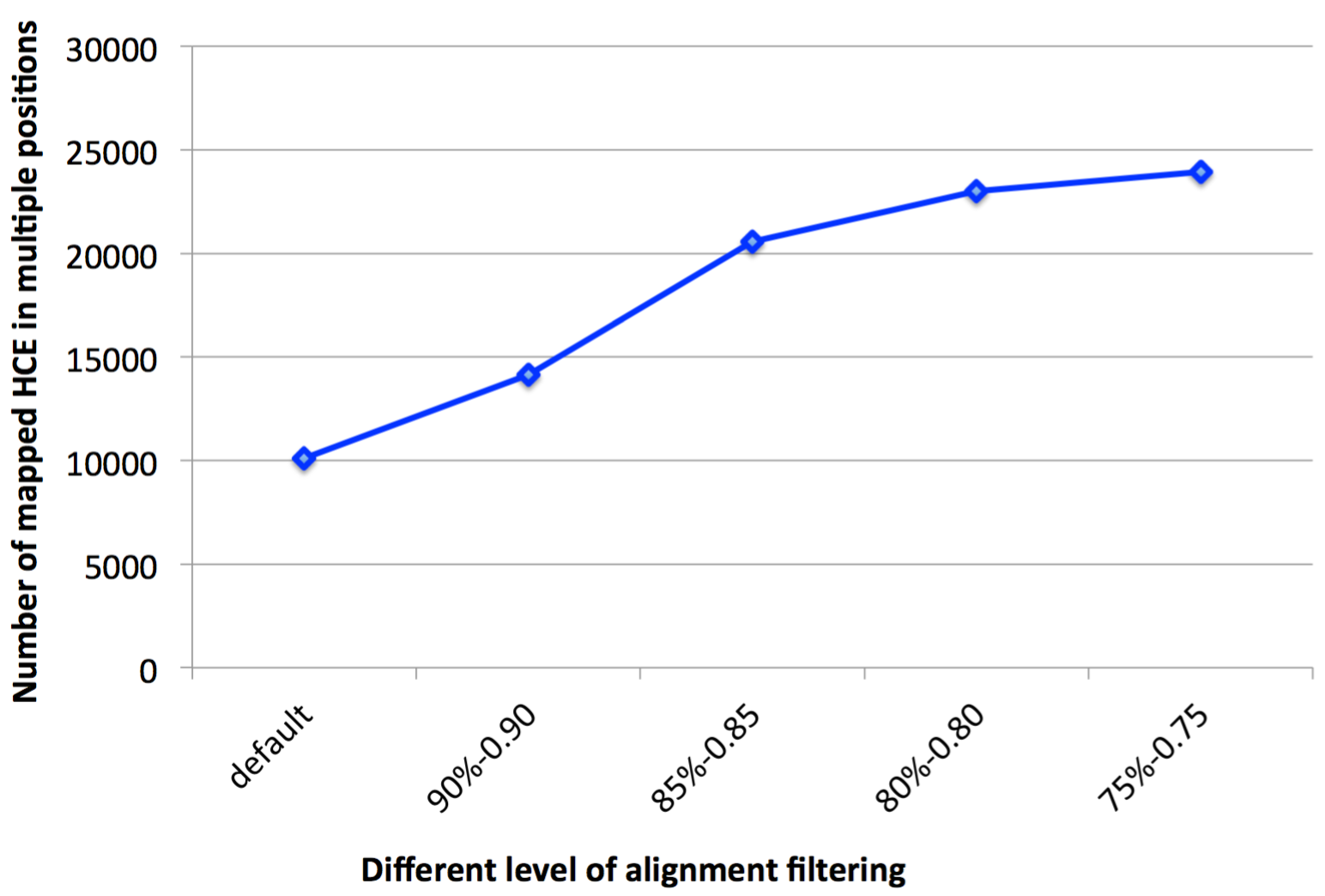


Figure 4: HCE that were mapped in multiple positions in Human-Mouse comparison. The relaxation of the HCE mapping criteria affected also the number of the HCE that were mapped in multiple positions.


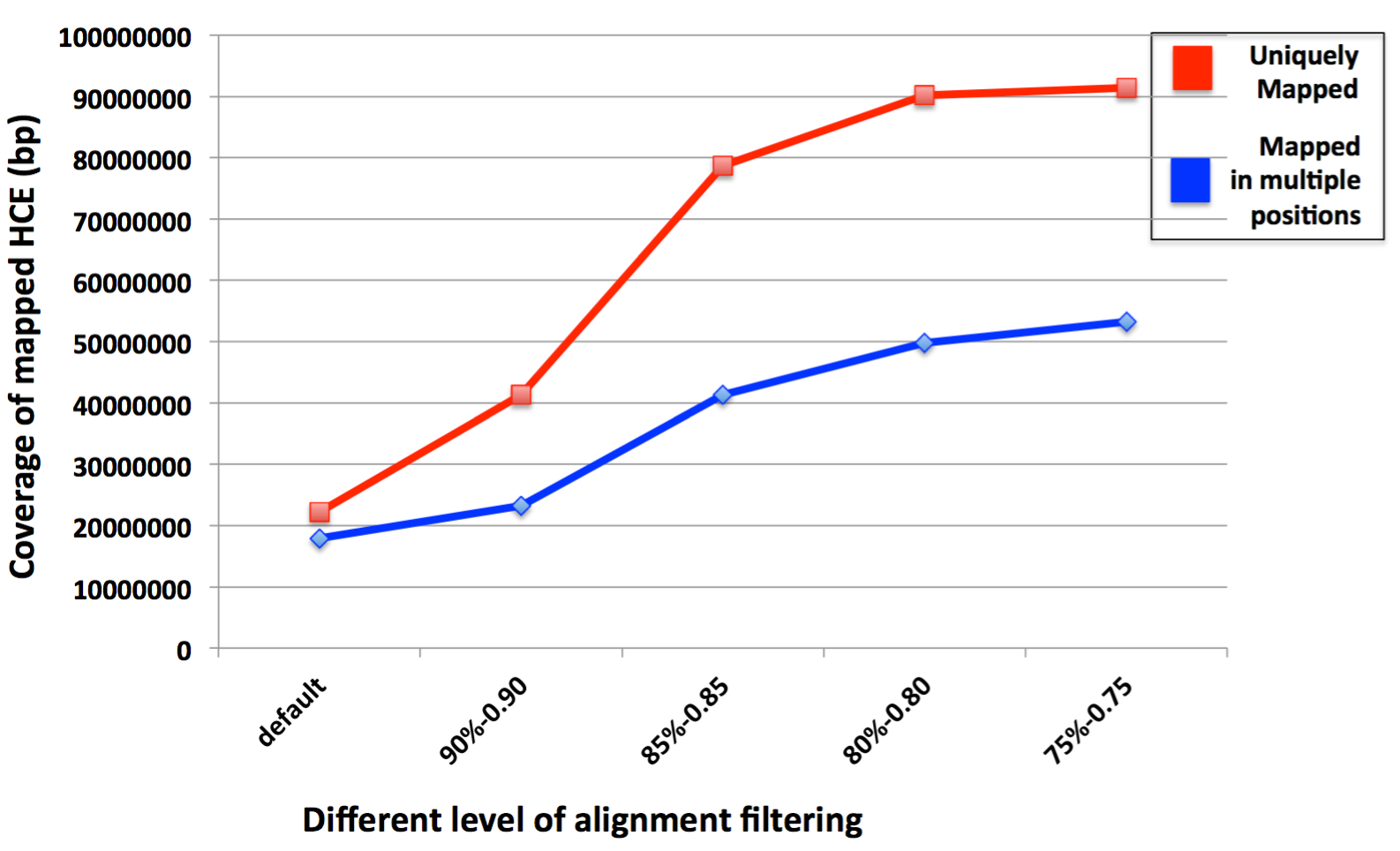


Figure 5: HCE that were mapped by using different filtering criteria (in terms of coverage). It is noticeable that by relaxing the filtering criteria of the anchoring construction, the total length that the HCE anchors cover on the ga-target increases sharply, comparing the increasing coverage of the HCE that were mapped on multiple positions.

**G-Anchor and Minimap: Times comparison**


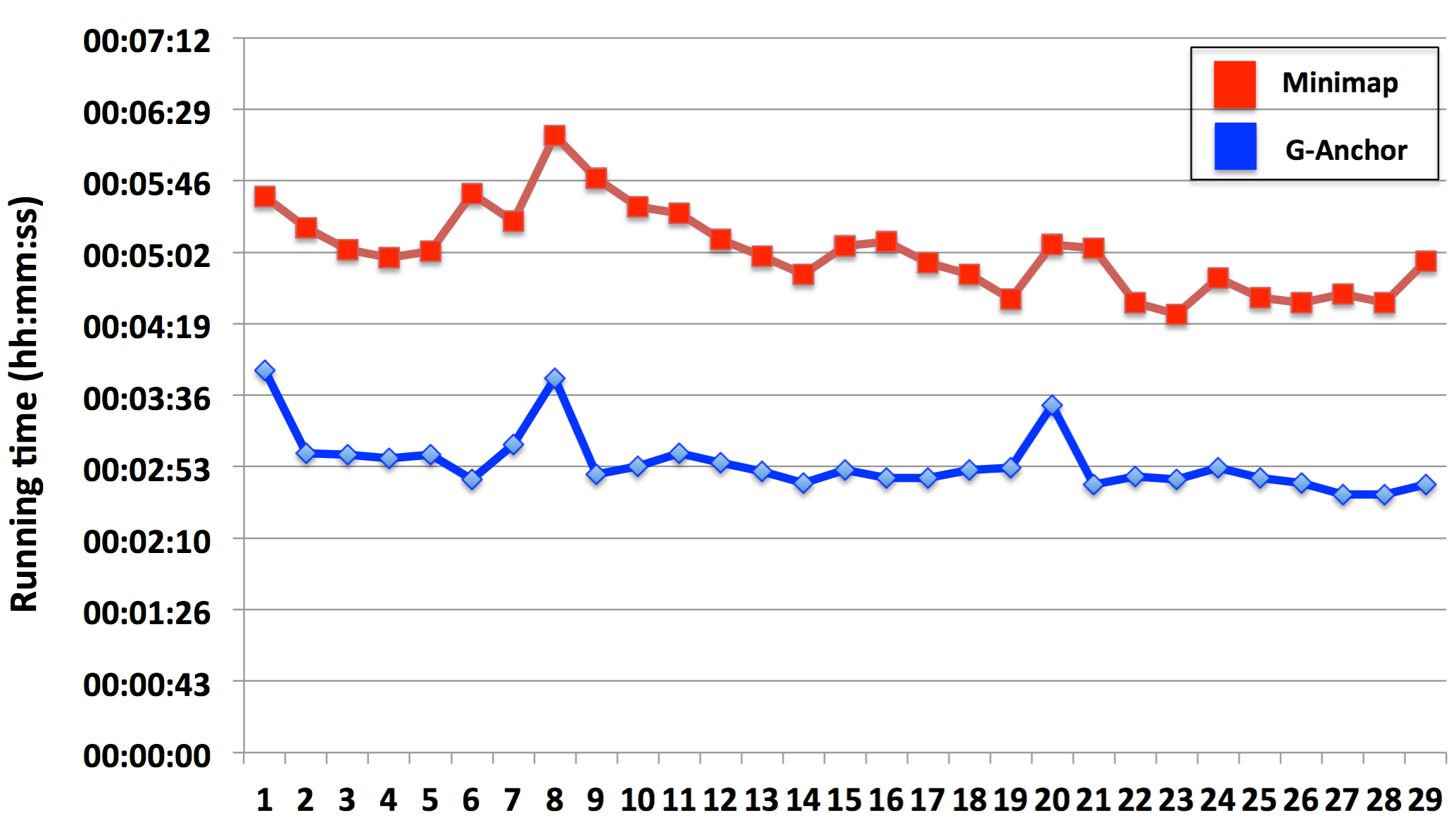


Figure 6: G-Anchor and Minimap running times. The 2 software was tested by using the Hum+Mam HCE databank for mapping Yak scaffolds to cattle autosomes. G-Anchor was faster as comparing with Minimap in all chromosomes.
