# Supplementary material for: G-Anchor: a novel approach for whole-genome comparative mapping utilizing evolutionary conserved DNA sequences
Source: Gigascience. 2018 Apr 3;7(5):giy017. doi: 10.1093/gigascience/giy017 (PMC5961299; doi:10.1093/gigascience/giy017)
Supplement: Response_to_Reviewer_Comments_Original_Submission.pdf [file giy017_response_to_reviewer_comments_original_submission.pdf]

Author's Response To Reviewer Comments

We thank for the reviewers for their further feedback. Our response to their suggestions is as follows.

Reviewer 1:
To more easily compare the run-time metrics of LASTZ, MUMmer, and G-Anchor, please report how long a whole genome alignment of the yak and cattle genomes takes using MUMmer 4.0. Please report in the same paragraph as LASTZ and MUMmer the total run-time of all stages of the G-Anchor pipeline for mapping the Yak genome to the Cow genome. Please report the multi-threaded wall-clock run-times of MUMmer and G-Anchor, noting that LASTZ is not multithreaded.

We have added this material between lines 505 and 544. Briefly:
" We give the time for LASTZ noting that is not multi-threaded (5 days, 4 hours).
" We report that MUMmer 4.0 takes 48 hours with a single core and 12 hours with 4 cores, and requires 43 GB RAM in both cases.
" G-Anchor requires 7 hours with a single core and 3 hours 15 minutes for 4 cores with 4.5 GB RAM. For the single core case, the times for the five stages are 4, 12, 11, 353, and 43 minutes respectively. Aligning the HCE with Blat takes the longest time: this was reduced from 353 (1 core) to 124 minutes (4 cores).

Reviewer 2:
Minor comments: 1. Combining math and (pseudo)code (e.g., Page 19 equation), while may serve the purpose of conveying the notion, may be written clearer using a standard pure math or pure algorithm notation.

We have added a standard pure math notation of the equation, between lines 436 to 444, as requested.

Editor:
In addition, please register any new software application in the SciCrunch.org database to receive a RRID (Research Resource Identification Initiative ID) number, and include this in your manuscript. This will facilitate tracking, reproducibility and re-use of your tool.

We have registered the software and added the RRID number (SCR_016046) at line 666.
