# Supplementary material for: G-Anchor: a novel approach for whole-genome comparative mapping utilizing evolutionary conserved DNA sequences
Source: Gigascience. 2018 Apr 3;7(5):giy017. doi: 10.1093/gigascience/giy017 (PMC5961299; doi:10.1093/gigascience/giy017)
Supplement: Reviewer_1_Report_(Original_Submission) -- Shaun Jackman — 13 Dec 2017 Reviewed [file giy017_reviewer_1_report_(original_submission).pdf]

Reviewer Report

Title: G-Anchor: a novel approach for whole-genome comparative mapping utilising evolutionary conserved DNA sequences

Version: Original Submission **Date:** 13 Dec 2017

Reviewer name: Shaun Jackman

Reviewer Comments to Author:

The authors have addressed the majority of my previous comments with their revised manuscript. Their concept of using precomputed evolutionarily conserved elements as seeds for mapping is new to me. Their tool outputs whole genome maps in a format suitable for viewing in the UCSC genome browser.

They have compared their whole-genome mapping tool G-Anchor to LASTZ and MUMmer. The alignment of two genomes using LASTZ took five days and 40 GB of RAM. The alignment of one chromosome using MUMmer 4.0 took 40 minutes and 43 GB of RAM. The computationally intensive step of their tool G-Anchor, alignment using BLAT, took 7 hours and 4.5 GB of RAM, a tenth of the memory, suitable for use on a workstation.

To more easily compare the run-time metrics of LASTZ, MUMmer, and G-Anchor, please report how long a whole genome alignment of the yak and cattle genomes takes using MUMmer 4.0. Please report in the same paragraph as LASTZ and MUMmer the total run-time of all stages of the G-Anchor pipeline for mapping the Yak genome to the Cow genome. Please report the multi-threaded wall-clock run-times of MUMmer and G-Anchor, noting that LASTZ is not multithreaded.

My best regards,
Shaun Jackman

Level of Interest

Please indicate how interesting you found the manuscript: An article of limited interest

Quality of Written English

Please indicate the quality of language in the manuscript: Acceptable

Declaration of Competing Interests

Please complete a declaration of competing interests, considering the following questions:

- Have you in the past five years received reimbursements, fees, funding, or salary from an organisation that may in any way gain or lose financially from the publication of this manuscript, either now or in the future?
- Do you hold any stocks or shares in an organisation that may in any way gain or lose financially from the publication of this manuscript, either now or in the future?
- Do you hold or are you currently applying for any patents relating to the content of the manuscript?
- Have you received reimbursements, fees, funding, or salary from an organization that holds or has applied for patents relating to the content of the manuscript?
- Do you have any other financial competing interests?
- Do you have any non-financial competing interests in relation to this paper?

If you can answer no to all of the above, write 'I declare that I have no competing interests' below. If your reply is yes to any, please give details below.

I declare that I have no competing interests.

I agree to the open peer review policy of the journal. I understand that my name will be included on my report to the authors and, if the manuscript is accepted for publication, my named report including any attachments I upload will be posted on the website along with the authors' responses. I agree for my report to be made available under an Open Access Creative Commons CC-BY license (http://creativecommons.org/licenses/by/4.0/). I understand that any comments which I do not wish to be included in my named report can be included as confidential comments to the editors, which will not be published.

I agree to the open peer review policy of the journal

To further support our reviewers, we have joined with Publons, where you can gain additional credit to further highlight your hard work (see: https://publons.com/journal/530/gigascience). On publication of this paper, your review will be automatically added to Publons, you can then choose whether or not to claim your Publons credit. I understand this statement.

Yes
